# Reactive OFF-ON type alkylating agents for higher-ordered structures of nucleic acids

**DOI:** 10.1093/nar/gkz512

**Published:** 2019-06-12

**Authors:** Kazumitsu Onizuka, Madoka E Hazemi, Norihiro Sato, Gen-ichiro Tsuji, Shunya Ishikawa, Mamiko Ozawa, Kousuke Tanno, Ken Yamada, Fumi Nagatsugi

**Affiliations:** 1 Institute of Multidisciplinary Research for Advanced Materials, Tohoku University, 2-1-1 Katahira, Aoba-ku, Sendai, Miyagi 980-8577, Japan; 2 Department of Chemistry, Graduate School of Science, Tohoku University, Aoba-ku, Sendai 980-8578, Japan

## Abstract

Higher-ordered structure motifs of nucleic acids, such as the G-quadruplex (G-4), mismatched and bulge structures, are significant research targets because these structures are involved in genetic control and diseases. Selective alkylation of these higher-order structures is challenging due to the chemical instability of the alkylating agent and side-reactions with the single- or double-strand DNA and RNA. We now report the reactive OFF-ON type alkylating agents, vinyl-quinazolinone (VQ) precursors with a sulfoxide, thiophenyl or thiomethyl group for the OFF-ON control of the vinyl reactivity. The stable VQ precursors conjugated with aminoacridine, which bind to the G-4 DNA, selectively reacted with a T base on the G-4 DNA in contrast to the single- and double-strand DNA. Additionally, the VQ precursor reacted with the T or U base in the AP-site, G-4 RNA and T-T mismatch structures. These VQ precursors would be a new candidate for the T or U specific alkylation in the higher-ordered structures of nucleic acids.

## INTRODUCTION

DNA is the most fundamental genetic material organized around the duplex structure to store and pass on genetic information. DNA and RNA can also adopt a significant number of varieties of stable higher-ordered structure motifs, such as the G-quadruplex, mismatch and bulge. Many of these secondary structures were found to be closely related to the control of the gene expression and the onset of critical diseases, therefore, becoming compelling therapeutic targets (1–4).

The G-quadruplex (G-4) is the subject of intensive study as a target for small molecule therapeutic intervention (5). For example, G-4 forming sequences have been found in the genomes of a variety of oncogene promoters such as *c-MYC* and *c-KIT*. In addition, G-4 is located at the end of the chromosome called a telomere, and known to be shortened each time a cell proliferates. When the telomere reaches a critical length, the cellular senescence is triggered. In contrast, most cancer cells significantly express telomerases to counteract the telomere shortening which is the basis of cancer cell proliferation and immortalization. Stabilization of the G-4 within the telomeric sequence by small molecules has been accepted as one of the strategies to inhibit this telomerase activity (6).

The study of triplet repeat diseases revealed their implication of mismatched structures (2) For instance, myotonic dystrophy type 1 (DM1) is one of the most common inherited diseases caused by continuous expansion of the simple trinucleotide repeats (CTG)_n_ and (CUG)_n_ sequences. It has been suggested that these abnormal CTG and CUG repeat sequences can form the T–T and U–U mismatches, respectively. The produced CUG repeat structures sequester the alternative-splicing regulator muscleblind-like proteins (MBNL) and prevent them from performing normal cellular activity that then leads to DM1. Thus, targeting these mismatches by small molecules will free the sequestered MBNL, thus serving as a promising therapeutic strategy (7–13).

**Figure 1.**
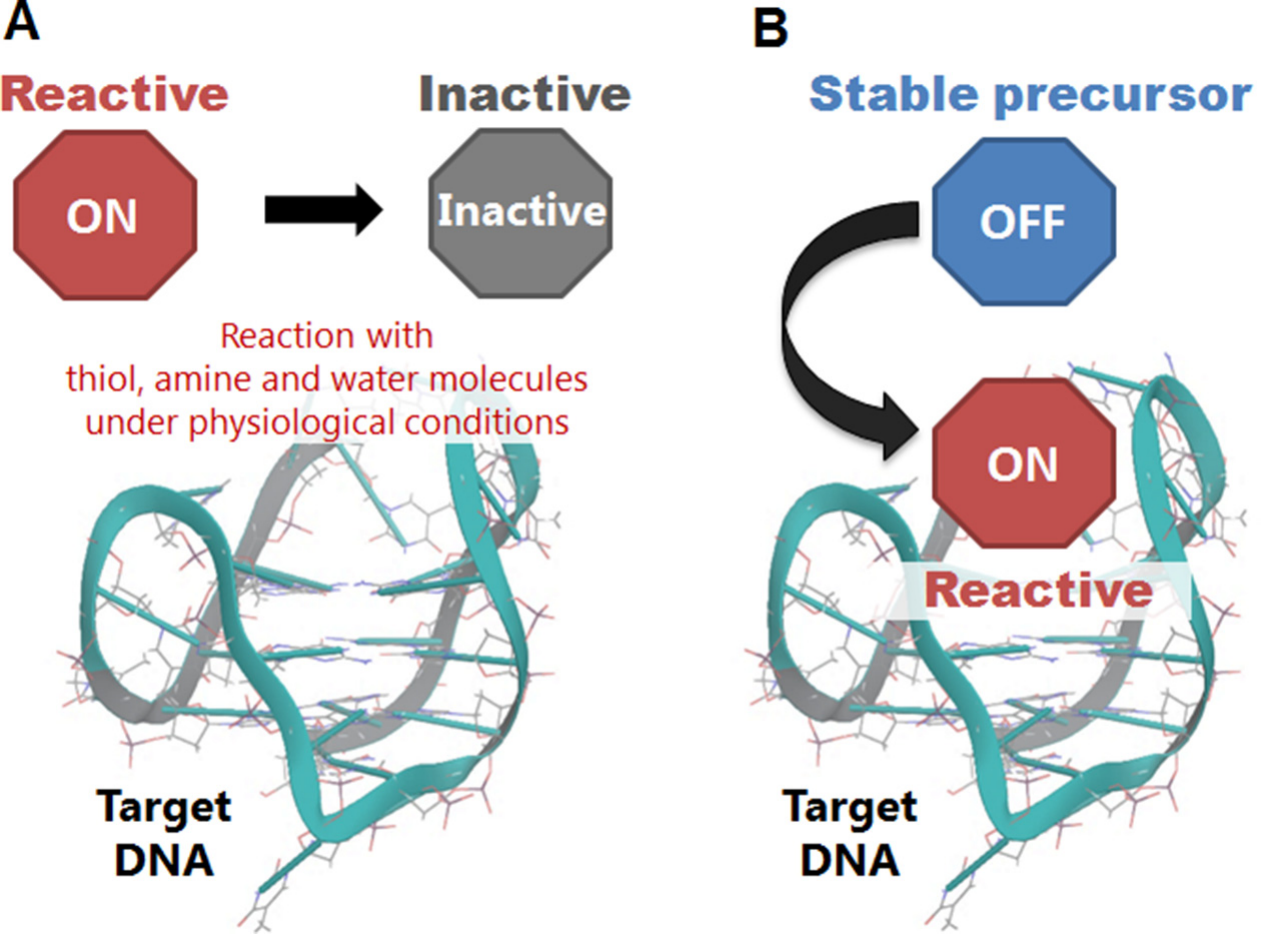
Schematic representation of the ligand inactivation under physiological conditions (**A**) and ligand-binding activation (**B**).

Due to the therapeutic potentials, efforts on the development of small molecule binders to specifically target the higher-order structures of nucleic acids have been intensely carried out. With the aim to augment the stabilization effect, selective alkylation using small molecules targeting the higher-order structures of nucleic acids has also been pursued, (12,14–19) However, most of the alkylating agents are facing a great concern about their effectivity and selectivity under physiological conditions. The abundant presence of biological species, such as thiol, amine, and water, would react with the reactive alkylating agent and prevent it from reaching and reacting with the target DNA or RNA (Figure 1A). Therefore, an improvement in the alkylating agent is required through establishment of a concept in which the alkylating ligand can remain in a stable OFF state until its alkylating reactivity is triggered at the target site only to then promote alkylation in a selective and effective manner (Figure 1B).

The reactive OFF-ON type alkylating agents, which are deprotected or activated by external stimuli, such as photo-irradiation or a chemical reagent, are some of the promising candidates to overcome the problems. For example, for the G-4 alkylation, benzophenone, aryl azide, the phenoxyl radical and the ruthenium complex were utilized as a photo-reactive group (20–22). The unique example of the thermally-induced G-4 alkylation using quinone methide precursors was also reported (23). As for other secondary structures, a chemical reagent, such as a fluoride ion (24) or rhodium, was utilized to induce alkylation (25). However, the alkylating agent activated just by binding with the target nucleic acid with no requirement of external stimulation has not yet been explored. We aimed to develop the OFF-ON type alkylating agents, which are activated only by binding to the target higher-ordered structures of nucleic acids without any external stimuli.

**Figure 2.**
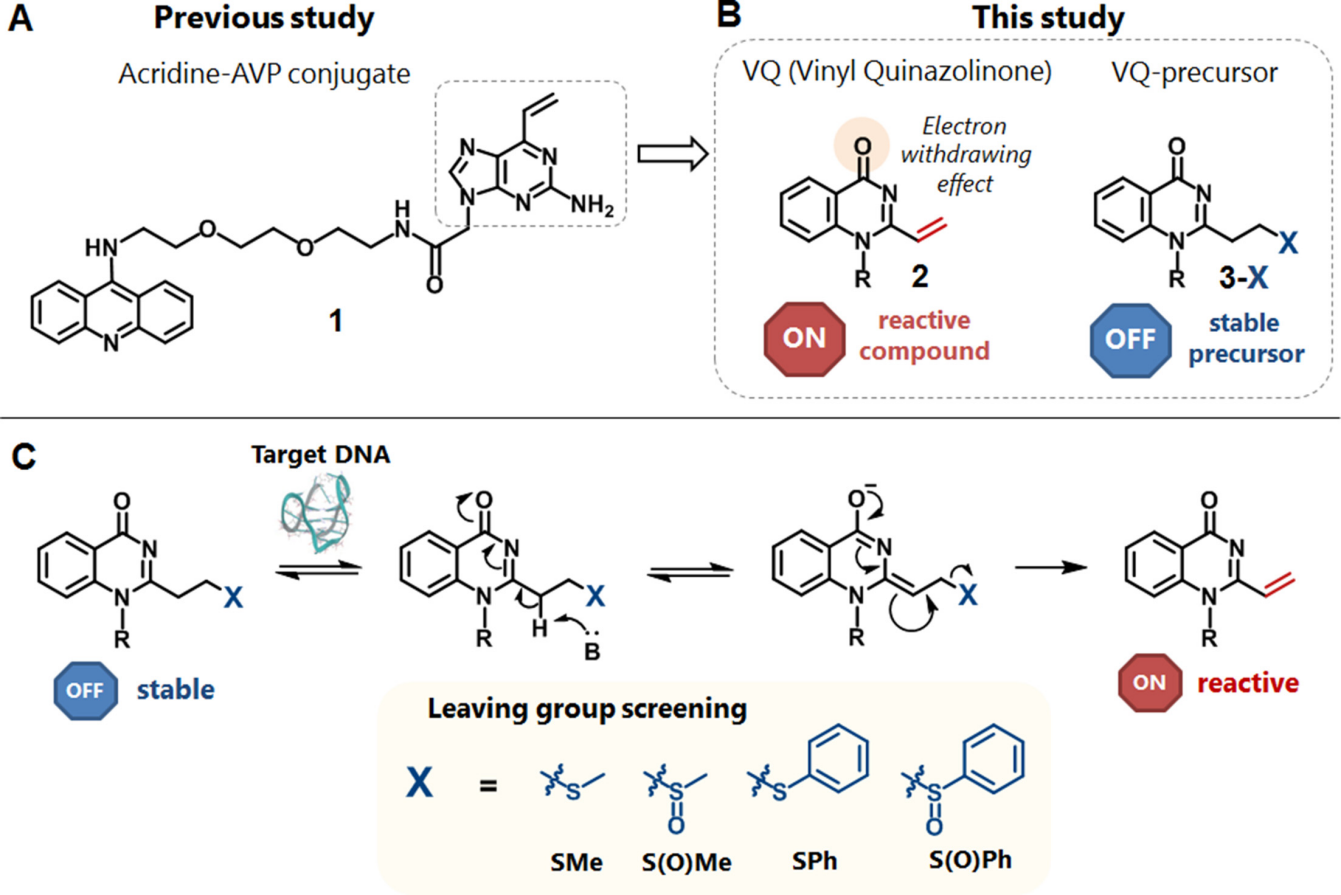
Molecular design. (**A**) The structure of acridine-AVP (2-amino-6-vinylpurine) conjugate (**1**) as the G-4 alkylating agent reported in a previous study. (**B**) The structure of acridine-VQ (vinylquinazolinone) conjugate (**2**) and its precursor (**3-X**) designed in this study. (**C**) The schematic concept of E1cB-type activation of VQ precursor (**3-X**). The first candidates of VQ-precursors; VQ-thiomethyl (**3-SMe**), VQ-methylsulfoxide (**3-S(O)Me**), VQ-thiophenyl (**3-SPh**) and VQ-phenylsulfoxide (**3-S(O)Ph**) precursors.

We previously developed the vinyl derivatives, 2-amino-6-vinylpurine (AVP) (Figure 2A) and vinyldiaminotriazine (VDAT), as G-4 and T-T mismatch alkylating agents, respectively (26–28). These acridine conjugates demonstrated the noteworthy ability to exclusively perform alkylation with the thymidine on the loop of the G-4 and T-T mismatch in a readily initiated and mild manner. Since the nucleophilic part of the thymidine *N*3 position is generally not accessible in the duplex structure, these ligands achieved a significant alkylating selectivity with the secondary structures. To develop the OFF-ON type alkylating agent, we retraced our oligonucleotide alkylation chemistry. The stable precursors of AVP, the phenylsulfide and phenylsulfoxide precursors, in the oligonucleotide, showed an effective cross-linking reactivity through the hybridization-promoted activation to the target DNA (29,30). Our experimental results suggested that the inducible reactivity of these stable precursors would be realized by the elimination of the sulfide or sulfoxide group and the resulting vinyl group generation through the binding to the target base in the duplex. In this study, we took advantage of this reactivity and induced-ability in this vinyl chemistry for the highly-selective alkylation of the higher-ordered structures of the nucleic acids.

## MATERIALS AND METHODS

The general chemicals were purchased from Fujifilm Wako Pure Chemical, the Tokyo Chemical Industry, Kanto Chemical or Aldrich. The target DNAs and RNAs were purchased from JBioS (Japan) (Table 1). The ^1^H NMR spectra (400 MHz) were recorded by a Bruker 400 spectrometer. The ^1^H NMR spectra (600 MHz) and ^13^C NMR spectra (150 MHz) were recorded by a Bruker AVANCE III 600 spectrometer. The high resolution electrospray mass analysis was performed by a Bruker MicrOTOFQ II. The HPLC purification was performed by a JASCO HPLC System (PU-2089Plus, UV-2075Plus, FP-2015Plus and CO-2065Plus). A reverse-phase C_18_ column (COSMOSIL 5C_18_-AR-II, Nacalai tesque, 4.6 × 250 mm or 10 × 250 mm for ligand purification). MALDI-TOF MS measurements were performed by a Bruker Autoflex speed instrument using a 3-hydroxypicolinic acid/diammonium hydrogen citrate matrix.

**Table 1.** ODN and ORN sequences used in this study

entry	sequences (5′-3′)
ODN**1**	FAM-TAGGGTTAGGGTTAGGGTTAGGG
ODN**2**	FAM-GCGCTGCCAG
ODN**3**	Alexa-CTGGCAGCGC
ODN**4**	TAGGGTTAGGGTTAGGGTTAGGGCAGAGAG
ODN**5**	FAM-CTCTCTGCC
ODN**6**	FAM-CTCTCTGCCCT
ODN**7**	FAM-CAGCGC**N**AATTCGCGAGT (**N** = T, C, A, G)
ODN**8**	ACTCGCGAATT**X**GCGCTG (**X** = dSpacer)
ODN**9**	Alexa-CTGGCTGCGC
ORN**1**	FAM-CAGCGC**U**AAUUCGCGAGU
ORN**2**	FAM-UAGGGUUAGGGUUAGGGUUAGGG
ORN**3**	FAM-GCGCUGCCAG
ORN**4**	Alexa-CUGGCUGCGC

### Alkylation of targets under neutral conditions

A solution (10 μl) of the VQ-precursor ligand (50 μM) and DNA [G-4, ds, ss, T-T, U-U or AP-site] (2.5 μM) in phosphate buffer (pH 7.0, K^+^ or Na^+^), [for G-4, ds, ss]; or in 50 mM MES buffer (pH 7.0) containing 100 mM NaCl [for T-T, U-U, or AP-site] (containing 2% DMSO) was incubated at 37°C. Aliquots (1.5 μl) of the reaction mixture were collected at various points of time and quenched by the addition of a loading buffer (3.0 μl, 95% formamide, 50 mM ethylenediaminetetraacetic acid (EDTA) pH 8.0, 0.05% bromophenol blue (BPB), 0.05% xylene cyanol FF) and the mixture was cooled to 0°C. Polyacrylamide gel electrophoresis (PAGE) was performed by a 16% polyacrylamide gel electrophoresis containing 20% formamide with 1 × TBE and 6.0 M urea at 500V for 90 min [for G-4], 60 min [for AP-site] and 30 min [for ds, ss, T-T and U-U]. The fluorescence labeled DNAs were visualized and quantified with FLA-5100 (Fujifilm Co., Tokyo, Japan). For the **3-S(O)Me** and **3-S(O)Ph** precursors, the ligands were used for alkylation without purification after the MMPP oxidation.

## RESULTS AND DISCUSSION

### Molecular design and synthesis of VQ-precursors

In an attempt to develop a novel agent with a high stability in a biological system and provide an effective and site-selective alkylation with the target structure, we developed the AVP alkylating moiety (**1**) to the vinyl-quinazolinone (VQ, **2**) (Figure 2). Due to its electron-withdrawing carbonyl group conjugated to the vinyl group, the vinyl group was expected to be highly reactive. To preserve the ligand reactivity only for the target site, we protected the highly-reactive vinyl group in VQ(**2**) with several functional groups (X), creating VQ-X precursors (**3-X**) (Figure 2B). We expected that the leaving group X detachment occurs by means of an E1cB-type elimination only when the ligand reaches and interacts with the target nucleic acids (Figure 2C) (31). The activation is carried out by an available proximate nucleobase and phosphate backbone to which the ligand is binding. The generated reactive VQ moiety will then promote the efficient alkylation with the target base. In order to address this aim, we have chosen various thiol and sulfoxide groups as the first candidates of the leaving group X, producing four different precursors, i.e., the VQ-SMe (**3-SMe**), VQ-S(O)Me (**3-S(O)Me**), VQ-SPh (**3-SPh**) and VQ-S(O)Ph (**3-S(O)Ph**) precursors (Figure 2C). The VQ precursors were conjugated with the aminoacridine as a general binder to the higher-ordered structures, since the contiguous binding of these ligands to the target structures would be necessary for the activation of these stable precursors.

**Scheme 1.**
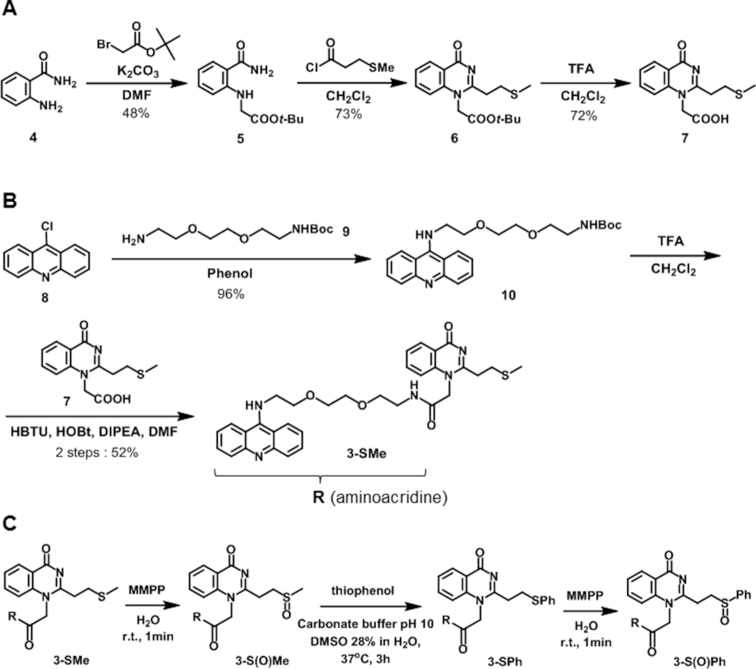
Synthesis of acridine-VQ-X precursors. (**A**) The carboxylic acid coupling unit of the VQ-SMe precursor (**7**). (**B**) Acridine-VQ-SMe precursor (**3-Me**). (**C**) Acridine-VQ-X precursors.

The preparation of the aminoacridine-VQ-X (**3-X**) is summarized in Scheme 1. The synthesis started with alkylation of the 2-aminobenzamide (**4**) starting material with *tert*-butylbromoacetate to give compound **5**. Cyclization of **5** with 3-methylthiopropionylchloride followed with *t-*Bu deprotection by TFA was carried out to give the carboxylic acid coupling unit of the stable VQ-SMe precursor (**7**). The aminoacridine binding moiety of **3-X** was prepared from 9-chloroacridine (**8**). Coupling of the monoBoc-protected amine linker (**9**) gave compound**10**. Compound **10** was then treated with TFA to deprotect the Boc group, then subsequent amidation with the VQ carboxylic acid coupling unit (**7**) gave the aminoacridine-VQ-SMe precursor (**3-SMe**). For the conversion to the other precursors, the SMe precursor (**3-SMe**) was oxidized by MMPP for 1 min to give the methylsulfoxide precursor (**3-S(O)Me**). A part of the isolated **3-S(O)Me** was spontaneously converted to VQ (**2**). The sulfoxide precursor was then treated with an excess amount of thiophenol, then incubated at 37°C for 3 h to give the VQ-thiophenyl precursor (**3-SPh**). Additionally, the **3-SPh** was converted to the phenylsulfoxide precursor (**3-S(O)Ph**) by oxidation with MMPP. These conversions were confirmed by HPLC and ESI-MS (Supplementary Figure S1). **3-S(O)Ph** was detected as an OH adduct due to its instability during HPLC. All of the VQ-X precursors were employed for the alkylation reaction with the DNAs or RNAs.

### Alkylating reactivity and selectivity of aminoacridine-VQ precursors

**Figure 3.**
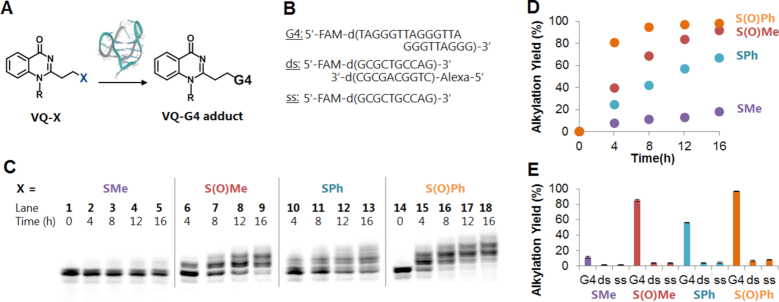
Screening of the leaving group for the G-4 alkylation. (**A**) Alkylation by acridine-VQ-X precursors. The precursor ligand (50 μM) was incubated with G-quadruplex (G-4), double-stranded (ds) or single-stranded (ss) DNA (2.5 μM) at 37°C in buffer solution (2% DMSO). G-4 phosphate buffer (K^+^): 100 mM KCl, 10 mM K_2_HPO_4_/ KH_2_PO_4_ (pH 7.0), 1 mM K_2_EDTA; ds and ss buffer: 100 mM NaCl, 10 mM Na_2_HPO_4_/NaH_2_PO_4_ (pH 7.0), 1 mM Na_2_EDTA. (**B**) The target sequences. (**C**) Denaturing gel electrophoresis of the alkylation products for G-4. Lanes 1 and 14 are for control (G-4 DNA without ligand). The electrophoresis was performed on a 16% denaturing polyacrylamide gel containing 20% formamide. (**D**) Time course of the reaction yields for **3-SMe, 3-S(O)Me, 3-SPh** and **3-S(O)Ph**. (**E**) Bar graph of yield (%) at 12 h for **3-SMe, 3-S(O)Me, 3-SPh** and **3-S(O)Ph** (*n* = 3, error bars indicate standard deviation).

We next assessed the ability of the aminoacridine-VQ conjugate precursors to form covalent adducts with several structures of DNA―(i) G-4 (ODN**1**), (ii) double-stranded DNA (ds, ODN**2**-ODN**3**) and (iii) single-stranded DNA (ss, ODN**2**) (Figure 3 and Table 1). We chose the human telomeric sequence as the G-4, folded in a K^+^ and Na^+^ containing buffer to give hybrid and anti-parallel topologies, respectively. The alkylating reaction was carried out by treating the target DNAs (2.5 μM) with 20 molar equivalents of each precursor (**3-X**) followed by incubation at 37°C. The **3-S(O)Me** and **3-S(O)Ph** precursors were used for the alkylation without purification after the MMPP oxidation. Analysis of the reaction mixture by denaturing PAGE showed the appearance of bands with a slower mobility attributed to the alkylated DNA products (Figure 3C and Supplementary Figure S2). For the **3-S(O)Me** and **3-S(O)Ph** precursors, clear bands indicating di- and tri-adducts were observed in addition to the mono-adduct.

The screening results showed that all the precursors formed covalent adducts with the G-4 DNAs, while almost no alkylation proceeded with the other targets, dsDNA and ssDNA. The comparison of the alkylating reactivity of each of the precursors with the G-4 could be clearly observed on the alkylation yield graph (Figure 3D). The phenylsulfoxide precursor (**3-S(O)Ph**) achieved the highest reactivity followed by **3-S(O)Me, 3-SPh** and **3-SMe** for the folded G-4 in both the K^+^ and Na^+^ buffers (Figure 3D and Supplementary Figure S2). The bar graph of the yield after a 12-h reaction time demonstrated the precursors’ great alkylation selectivity toward the secondary structure of G-4 over the dsDNA and ssDNA (Figure 3E). The graph has also clearly shown that the precursors have a preference to alkylate with the hybrid topology in the K^+^ buffer over the anti-parallel structure in the Na^+^ buffer (Supplementary Figure S2). The CD spectra indicated that the aminoacridine ligand binding did not change the G4 topology (Supplementary Figure S3). These results suggested that the position of the G4-loop affects the alkylation efficiency. In order to obtain insight into the mechanism of alkylation, we further investigated the adduct structure and alkylation site.

### Determination of the adduct structure and alkylation site

With the aim to determine the adduct structure, enzymatic hydrolysis of the alkylated oligodeoxynucleotide (ODN) was performed (Supplementary Figure S4). The mono-alkylated ODN was prepared with **3-SPh**. The alkylated ODN was subjected to enzymatic hydrolysis using alkaline phosphatase and phosphodiesterase I. The product was purified and analyzed by HPLC and ESI-MS (Supplementary Figure S4). In addition to the native nucleosides (dG, dT and dA) and fluorophore FAM, a single new peak VQ-dT* was observed in the HPLC profiles for both of the alkylated G-4s in the K^+^ and Na^+^ buffers. This peak was assigned by ESI-MS to be the thymidine adduct with one molecule of the aminoacridine-VQ ligand.

**Figure 4.**
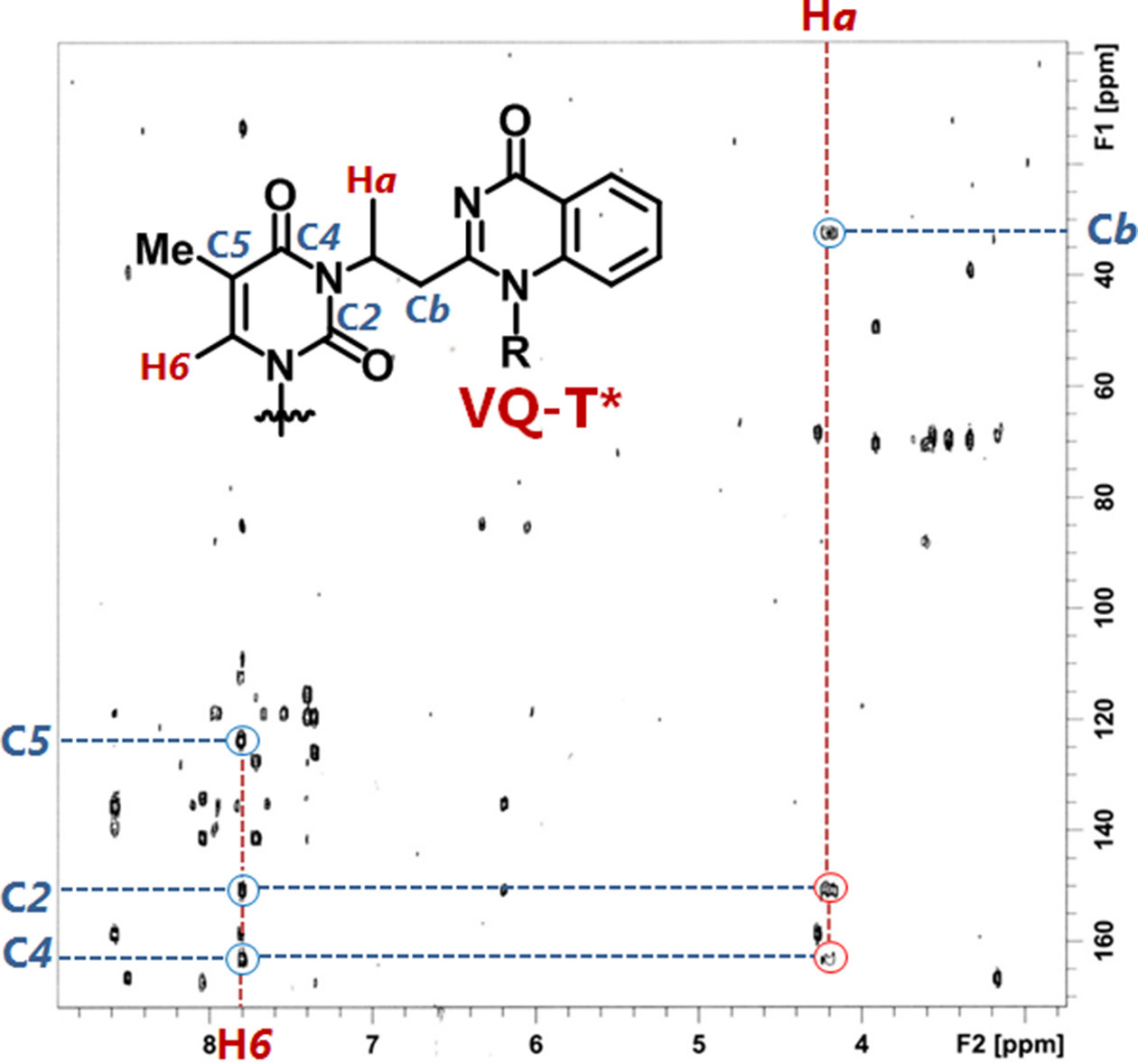
HMBC-NMR spectrum for the HPLC purified VQ-dT**. The spectra indicate resonance of one of the ethylene protons between thymidine and quinazolinone molecule with two carbons from thymidine.

In order to clearly determine the adduct position on the thymidine, we attempted to record the nuclear magnetic resonance (NMR) of the nucleoside adduct. To obtain a sufficient amount of the adduct products for the NMR measurements, we first performed the monomer reaction between the VQ-methylsulfoxide (**3-S(O)Me**) precursor and thymidine (Supplementary Figure S5). The reaction was carried out in DMSO under highly-concentrated conditions at 37°C for 3 days. The reaction analysis and product purification were carried out by HPLC (Supplementary Figure S5). The purified alkylated thymidine VQ-dT** ^1^H-NMR and HMBC NMR were then recorded and assigned to reveal the exact position of the adduct (Supplementary Figure S5). We previously reported the NMR spectra recorded for the vinyldiaminotriazine (VDAT) acridine conjugate reacted with the thymidine at the *N3* position (27). Referring to the recorded COSY, edited-HSQC and HMBC NMR from this particular previous report, we could then assign the position of the carbon at the thymidine's carbonyl group, C*2* and C*4* as well as the proton H*a* from one of the ethylene protons between the quinazolinone and thymine base. The recorded HMBC NMR for VQ-dT** gave correlation peaks between the H*a* and two carbon resonances at around 160 ppm, assigned as C*2* and C*4* (Figure 4). This result concluded that the position of the alkylation of VQ-dT** is the *N*3 position. This is due to the consideration that if the VQ-dT** alkylation was either at *O*2 or *O*4, these HMBC correlation peaks would not appear.

The last step was to determine whether the VQ-dT** adduct, whose structure was confirmed by NMR, is the same as the one obtained from the G-4 alkylation. Toward this aim, we carried out the enzymatic hydrolysis on a large amount of the alkylated G-4, performed an HPLC analysis and purification of the VQ-dT* adduct, then co-injected the purified product of VQ-dT* with the NMR-confirmed VQ-dT** (Supplementary Figure S5). The HPLC analysis result of the VQ-dT* and VQ-dT** co-injection suggested a clean single peak for both the alkylated G-4 in the K^+^ and Na^+^ buffers, concluding that the VQ ligand reacted with G-4 at the *N*3 position of the thymidine.

**Figure 5.**
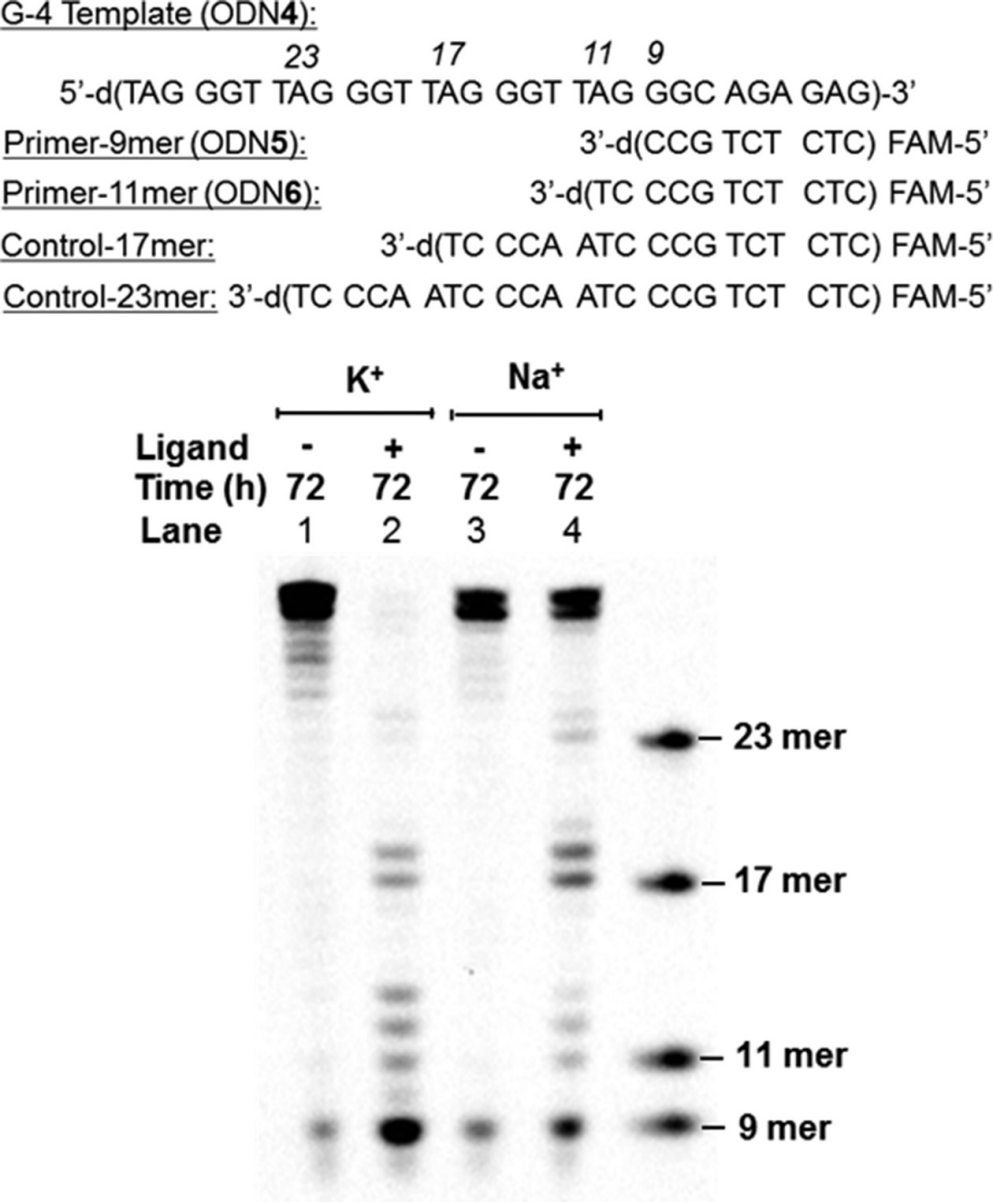
Primer extension assay of non-alkylated and alkylated G-4 DNA. Alkylation was carried out with the G-4-template ODN**4** (2.5 μM) and compound **3-SPh** (50 μM) in K^+^ buffer or Na^+^ buffer for 72 h. The alkylated template DNA was then annealed with primer-9 (ODN**5**). The primer extension reactions were performed by incubating the G-4 template (5 μM) and primer DNA (0.15 μM), Klenow Fragment (exo^−^) (0.1 U/μl) and dNTP (0.2 mM) in NEbuffer pH 7.9 at 37°C for 5 min.

An additional effort to further confirm the alkylation sites along the G-4 was carried out by a primer extension assay (Figure 5 and Supplementary Figure S6). To prepare the alkylated template DNA, the alkylation was first performed on the 30 mer G-4 template (ODN**4**) folded in the K^+^ or Na^+^ buffer with the ligand **3-SPh**. After the reaction, the reaction mixture was then annealed with a fluorescein (FAM) labeled primer of 9 mer (ODN**5**) or 11 mer (ODN**6**) length to be subjected to primer extension using the Klenow Fragment (exo-) DNA polymerase. The extended DNA fragments were visualized by fluorescence during the PAGE analysis (Figure 5 and Supplementary Figure S6). PAGE analysis of the primer extension of the alkylated G-4 (K^+^) template using the 9 mer primer (ODN**5**) gave the appearance of clear bands at the 11, 12, 13, 17, 18, 23 and 24 mer (Figure 5, lane 2). These bands were indicative of the alkylation at bases just next to the stopping bands, which were 12T, 13T, 14G, 18T, 19T, 24T and 25T, respectively. PAGE analysis of the alkylated G-4 (Na^+^) also indicated alkylation of the 20G in addition to those observed in lane 2 (Figure 5, lane 4). These results implied that alkylation exclusively occurred with the T base. The stop at the 14G and 20G positions might come from extension inhibition by the G-4 structure itself rather than the result of alkylation. There were no clear stopping band appearances on the lane of the non-alkylated template (Figure 5, lanes 1 and 3). Similar patterns of the bands were observed for the primer extension of the G-4 templates using the 11 mer primer (ODN**6**) (Supplementary Figure S6). From the difference in the stopping band ratio between the K^+^ and Na^+^ buffers, the preferred alkylation site would depend on the G4 topology. In our previous study, using the acridine-AVP and acridine-VDAT conjugates, similar results from the primer extension assay were obtained (26,28). These results validated that the VQ-ligand alkylation proceeds with the thymidine of the G4-loop.

### Activation mechanism of aminoacridine-VQ sulfoxide precursors

As already described, the VQ-sulfoxides demonstrated the highest reactivity among all the other precursors. In order to evaluate the stability of both precursors under physiological-like conditions, we treated **3-S(O)Me** and **3-S(O)Ph** with excess glutathione (GSH) and analyzed the formation of the GSH adduct by HPLC and ESI-MS (Supplementary Figure S7). For **3-S(O)Me**, the GSH-adduct gradually formed after an 8-h reaction time (Supplementary Figure S7B). Based on the comparison between the area of the newly formed GSH-adduct peak and the unreacted ligand peak of the **3-S(O)Me**, the reaction rate and ligand half-time (*t*_1/2_) were calculated. The reaction rate was 2.7 × 10^−5^ s^−1^ and the *t*_1/2_ of **3-S(O)Me** was 7.2 h. This result resonated well with the alkylation rate with G-4 that achieved around a 60% yield in 8 h. As for **3-S(O)Ph**, we observed the OH adduct peak on the HPLC profile instead of the **3-S(O)Ph** peak due to the instability. The clear GSH-adduct peak was observed in 2 h (Supplementary Figure S7C). Based on the collected results, we suggest that the precursors spontaneously convert into the reactive vinyl form under aqueous conditions with no requirement of any trigger, thus prone to undergo attack from available nucleophiles.

### Activation mechanism of aminoacridine-VQ thiophenyl precursors

**Figure 6.**
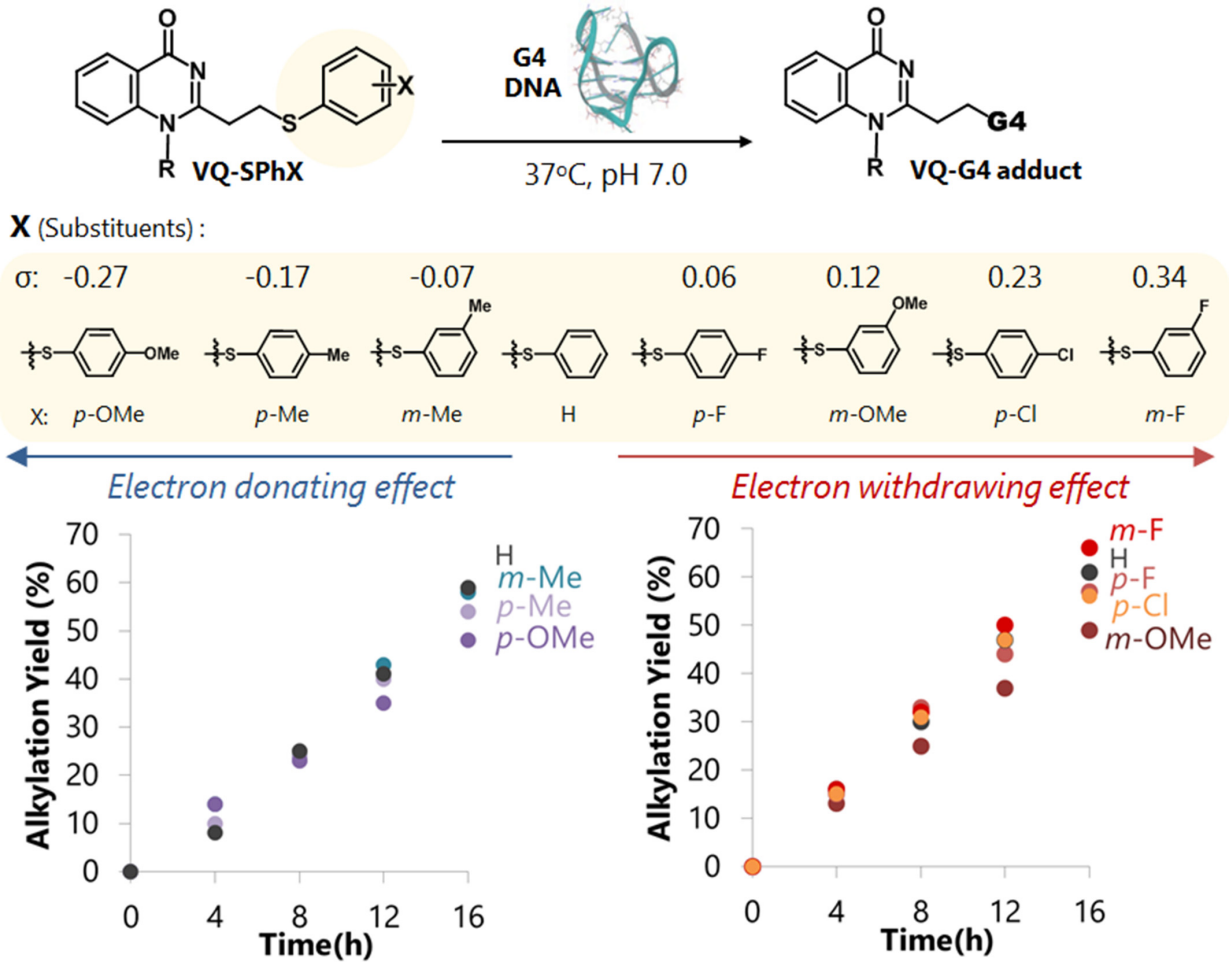
Alkylation reaction of VQ-SPhX with G-4 DNA. The precursor ligand (50 μM) was incubated with G-4 DNA folded in K^+^ (2.5 μM) at 37°C in buffer solution. G-4 potassium buffer (K^+^): 100 mM KCl, 10 mM K_2_HPO_4_/ KH_2_PO_4_ (pH 7.0), 1 mM K_2_EDTA. Time course of alkylation of VQ-SPhX with electron-donating effect (left) and with electron-withdrawing effect (right)

With the aim to further explore the potential of the thiophenol precursors, we attempted to prepare a series of thiophenol precursors having a variety of functional groups attached to the aromatic ring (Figure 6). We chose the methoxy (-OMe), methyl (-Me), chloro (-Cl) and fluoro (-F) groups attached at either the meta or para position to attempt different electronegativity levels on the thiophenyl leaving group. We expected that the thiophenol modified with electron withdrawing (EWG) substituents will result in a faster elimination step, thus increasing the alkylation efficiency. On the other hand, the leaving group modified with an electron donating group (EDG) will lead to a slower elimination rate and lower alkylation efficiency.

The synthesis of the aminoacridine-VQ with the thiophenol-derivative was carried out in a way similar to the synthesis of the thiophenol precursor. The starting compound, the thiomethyl precursor (**3-SMe**), was oxidized by MMPP to give the methylsulfoxide precursor (**3-S(O)Me**). The sulfoxide precursor was then treated with an excess amount of the substituted-thiophenol and then incubated at 37 or 45°C, depending on the substituents, for several hours to give the VQ in its substituted-thiophenol precursor (**3-SPhX**) (Supplementary Table S1). The conversion was traced and purified by HPLC and the compound products were determined by ESI-MS spectroscopy.

After the preparation of all the substituted-thiophenol precursors, we next investigated the alkylating reactivity of those precursors with the G-4 DNA folded in either the K^+^ or Na^+^ buffer. The alkylation reaction was carried out by incubation at 37°C of the mixture of G-4 DNA (2.5 μM) and 20 molar equivalents of the ligand. Analysis of the mixture by denaturing gel electrophoresis showed the formation of retarded bands which can be assigned as the alkylated G-4 products (Supplementary Figure S8). The calculated alkylation yields were then plotted to provide a reactivity comparison between the ligands as shown in Figure 6 and Supplementary Figure S9. To our surprise, the alkylation reactivity did not follow according to the electronegativity traits of the substituted-thiophenol leaving group. It was obvious from this result that a greater electronegative para-chlorothiophenol precursor (**3-SPh-*p*Cl**) produced a lower alkylation yield than the non-substituted thiophenol precursor (**3-SPh**) for both G-4 targets in the K^+^ and Na^+^ buffers.

**Figure 7.**
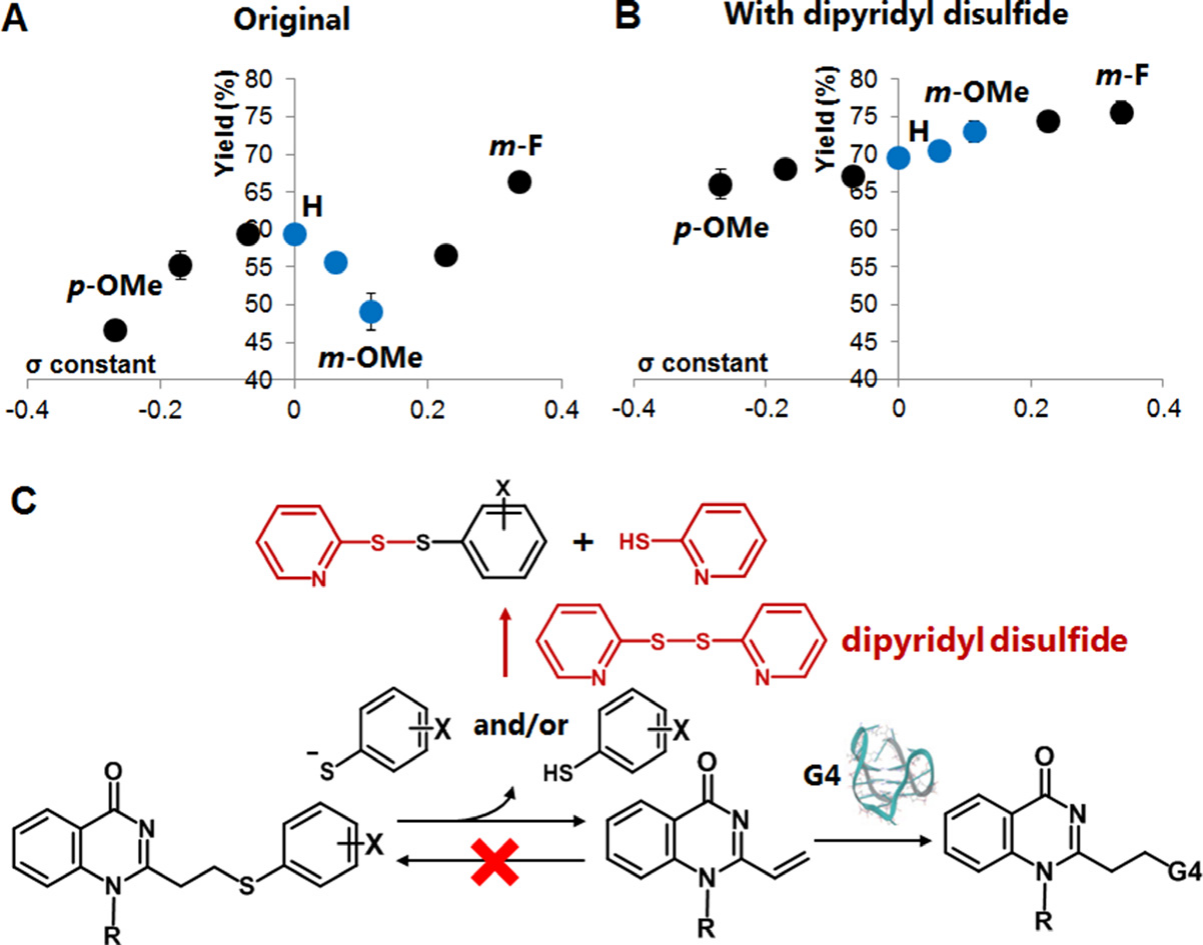
Relationship between Hammett constant and alkylation yield for VQ-SPhX precursors. (**A**) The reaction with G-4 in K^+^ buffer (*n* = 3, error bars indicate standard deviation). (**B**) Reaction in the presence of dipyridyl disulfide (*n* = 2). (**C**) The plausible vinyl generation mechanism of VQ-SPhX and thiol trap with dipyridyl disulfide.

To further understand the substituent effect, we plotted the reaction yields at 16 h versus the reported Hammett constant values of the substituted thiophenols (32). The plot was obtained for the target G-4 in the K^+^ (Figure 7) and Na^+^ buffers (Supplementary Figure S10). Both of the obtained plots obviously did not show the typical linear correlation; instead, they consisted of three phases (upward to the right: *p*-OMe to H and *m*-OMe to *m*-F, downward to the right: H to *m*-OMe). We focused our attention on the eliminated thiol state, molecular or ionic form or their mixture, and their backward reaction to understand the plot’s deviation. The electron donating group (EDG) substituents (*p*-OMe, *p*-Me and *m*-Me) defined the first positive-slope line. In this phase, the released thiophenols should favor the molecular form (Ar-SH), as their p*K*_a_ values are higher than the pH of the reaction solution. The second positive-slope line was observed for the electron withdrawing group (EWG) substituents (*m*-OMe, *p*-Cl and *m*-F). In contrast to the first positive slope, the eliminated thiophenol groups along this line have lower p*K*_a_ ‘s, thus they will be in their ionic form (Ar-S^−^). The ionic form of the thiol is much more nucleophilic than the molecular form, significantly promoting a fast backward reaction. As a consequence, the overall yields of the EWG-modified thiophenol were not significantly higher than the EDG-modified one, regardless of the fact that the EWG-substituted thiophenols have a better leaving ability than EDG. With these interpretations in mind, the negative slope line (blue dots in Figure 7A) can then be assigned to the transition phase from the molecular to ionic form (H, *p*-F and *m*-OMe). The amount of the eliminated ionic thiol increases along with the electronegativity increase of the substituents or along with the increase in the Hammett constant values. Since the ionic form induces the rapid backward reaction, the increase in the ionic form would result in a decrease in the overall yields, thus the negative slope.

In order to validate the interpretation, we stopped the backward reaction by the treatment of excess 2, 2’-dipyridyl disulfide (Figure 7B and C). Dipyridyl disulfide would trap the released substituted-thiophenols in both the molecular or ionic form, thus completely eliminating the backward reaction occurrence. Since the backward reaction is excluded, we can expect to observe a direct relation between the forward reaction and substituent’s electronegativity. It could be clearly observed from the result that the linear plot was obtained from the alkylation reaction performed in the presence of dipyridyl disulfide. In addition, the overall yields increased because the backward reaction was inhibited. This result strongly suggested that the significant deviations of these plots were attributed to the backward reaction effect, which is dependent upon the nature of the substituted thiophenol leaving group. The result has also suggested that vinyl generation of the VQ-thiophenol precursors took place in an equilibrium state.

As an additional validation effort, we attempted to perform an alkylation reaction under a different, slightly acidic pH condition. As can be seen, the first positive-slope line range widened as the downfall point shifted to a higher Hammett constant value or to a more EWG substituent (Supplementary Figure S11). This result further validated our assumption that the negative slope line as well as the deviation of the plot as a whole was indeed attributed to the ionic and molecular characters of the eliminated substituted thiophenyl group.

In order to further validate that the leaving groups were indeed in the form of a thiol, we performed a BES-Thio fluorescence assay. BES-Thio is a commercially-available fluorescent probe that selectively responds to the thiol under neutral conditions and thus is commonly used to measure the amount of thiols in a solution (33). Upon detection of the eliminated thiophenyl groups coming from the vinyl compound generation, the BES-Thio would release a fluorophore that emits a fluorescence which could be measured by fluorescence spectroscopy (Supplementary Figure S12A). As shown in Supplementary Figure S12B, an increase in the fluorescence intensity was clearly observed after 3 and 6 h of incubating the thiophenol precursor **3-SPh** with G-4 DNA and BES-Thio at 37°C. It can be seen that the incubation of only BES-Thio did not show any enhancement to the emission intensity. On the other hand, incubation of **3-SPh** with BES-Thio without G-4 led to a slight enhancement. These results clearly indicated that thiophenol was eliminated in an equilibrium state from **3-SPh**.

### Activation mechanism of aminoacridine-VQ thiomethyl precursor

**Figure 8.**
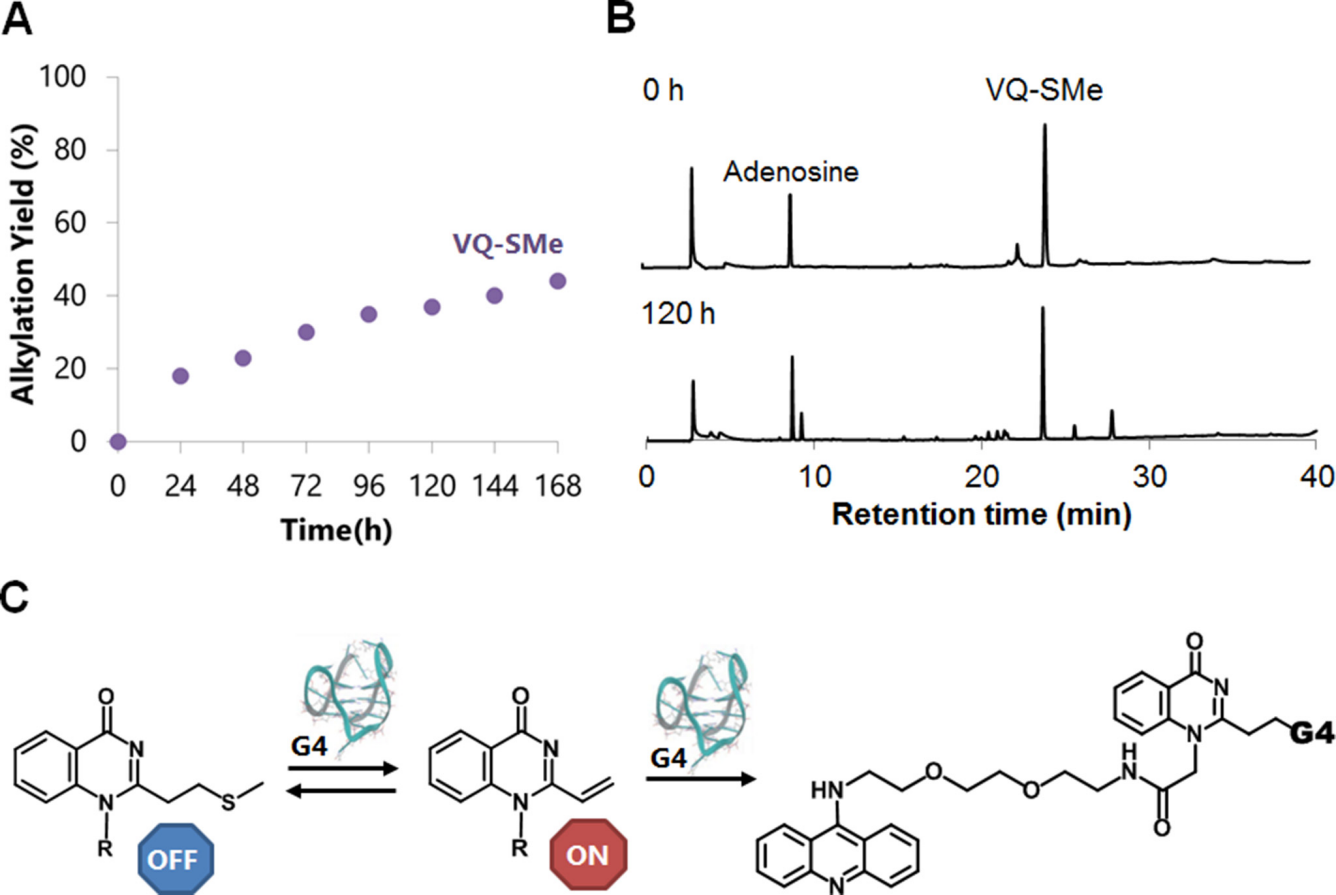
Alkylation by acridine-VQ-SMe precursor (**3-SMe**). The precursor (50 μM) was incubated with G-4 DNA (2.5 μM) at 37°C in buffer solution. G-4 potassium buffer (K^+^): 100 mM KCl, 10 mM K_2_HPO_4_/ KH_2_PO_4_ (pH 7.0), 1 mM K_2_EDTA. (**A**) Time course of the alkylation. (**B**) The HPLC profiles before reaction (top) and after 120-h reaction (bottom) with GSH (1 mM). Adenosine was added as an internal standard. (**C**) The plausible vinyl generation mechanism of VQ-SMe precursor. E1cB elimination will occur only in the presence of the G-4 DNA.

After gaining insights into the alkylation profiles and reaction mechanism of the VQ in the sulfoxide and thiophenol precursors, we evaluated the VQ-thiomethyl precursor. Among the four precursors, VQ-thiomethyl showed the lowest reactivity as shown in the Figure 3. To observe the reactivity halt, we attempted alkylation in a longer time frame (168 h) and analyzed the reaction by PAGE (Supplementary Figure S13). The obtained alkylation yields plot gave a clearer observation that the alkylation steadily proceeded in a moderate rate for up to 168 h (Figure 8A). The reaction with G-4 folded in the K^+^ buffer was preferred over the Na^+^ one by producing a higher overall yield (Supplementary Figure S13). We then assessed the precursor’s stability by presenting an excess nucleophile in a solution. We treated **3-SMe** with excess GSH, incubated the reaction mixture at 37°C for 120 h, then analyzed the formation of the GSH adduct by HPLC (Figure 8B). The HPLC analysis showed that **3-SMe** did not form an adduct with GSH and neither decomposed even after a long 120 h of incubation, demonstrating the impressive stability of the VQ thiomethyl precursor. These abilities of **3-SMe** led us to perceive that the elimination of the SMe group is triggered by the presence of G-4 as initially expected. The VQ-thiomethyl precursor (**3-SMe**) would undergo activation through the E1cB elimination that occurs when the precursor is in close proximity to the G-4 site, generating a vinyl that promotes reactivity with the target G-4 (Figure 8C).

### Aminoacridine-VQ precursor alkylation ability with other higher-ordered structures of nucleic acids

**Figure 9.**
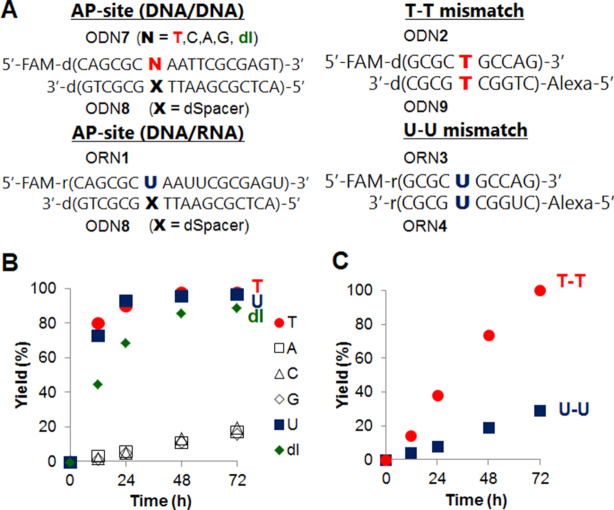
Alkylation with AP-site-containing DNA and T-T/U-U mismatch-containing DNA/RNA by acridine-VQ-SPh precursor (**3-SPh**). The precursor (50 μM) was incubated with target DNA or RNA duplex (2.5 μM) at 37°C in 50 mM MES buffer (pH 7.0) containing 100 mM NaCl. (**A**) The sequences of the target DNA or RNA. (**B**) Time course of the reaction yields for AP-site. (**C**) Time course of the reaction yields for T-T (circle) and U-U (square).

**Figure 10.**
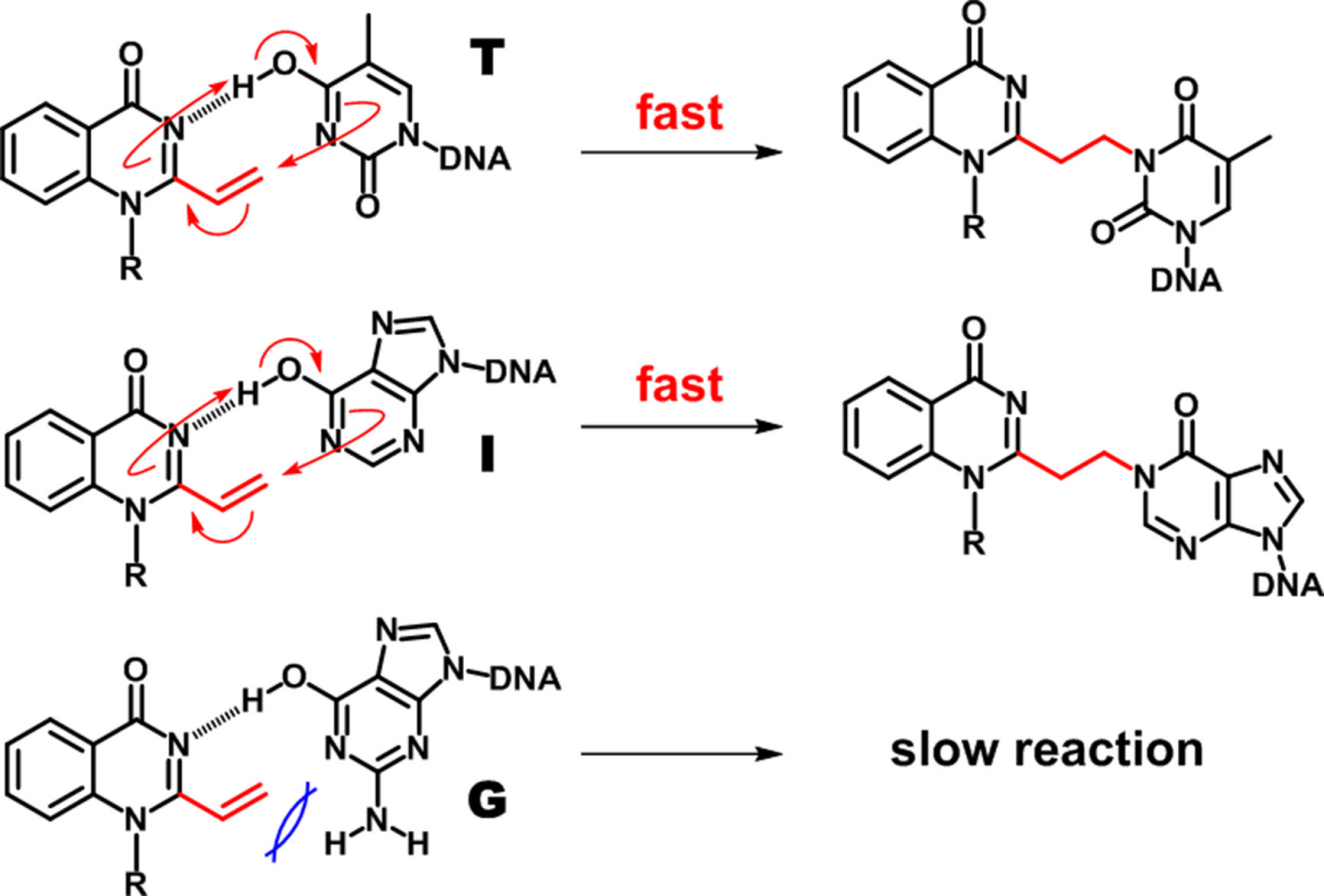
Plausible alkylation mechanism of VQ. The efficient alkylation to the thymidine (top) and inosine (middle). The slow alkylation to the guanosine base (bottom).

Knowing the potential of the VQ-precursor as a promising alkylating moiety, we were encouraged to utilize the compound to target other higher-ordered structures of nucleic acids such as an AP-site and T-T/U-U mismatch on DNA or RNA (Figure 9A). We have previously developed several 2-amino-6-vinylpurine (AVP)-conjugated ligands for the AP-site-targeting alkylation (34). The AP site allows a variety of ligands to recognize the site through hydrogen bonding in the generated hydrophobic pocket (35–41). The alkylation to the AP-site-containing duplex, ODN**7**(N = T, C, A, G)-ODN**8**, was attempted by the aminoacridine-VQ-SPh precursor (**3-SPh**). Interestingly, the alkylation mostly proceeded only to the thymidine at the AP-site (Figure. 9B and Supplementary Figure S14). These results demonstrated the potential of the VQ-precursor as a potent alkylating moiety that can target a variety of structures in an impressive and selective manner toward the thymidine. Additionally, the alkylation smoothly proceeded to the deoxyinosine (Figure 9B and Supplementary Figure S14). Given that VQ alkylates the thymidine at the *N*3 position, the 4-pyrimidinone structure is essential to efficiently alkylate the target by the VQ. We suggest a possible alkylation mechanism to explain the selectivity and efficiency to the thymine base. As illustrated in Figure 10, we speculated a concerted mechanism in which the VQ reacts with the enol form of the thymidine or inosine base. The proton transfer from the target base to the VQ may be a key factor for the efficient alkylation. The guanosine base would not react due to the steric hindrance of the amino group or lower reactivity.

To confirm the reactivity with uridine in the RNA substrate, the alkylation to the AP-site-containing DNA/RNA duplex (ORN**1**-ODN**8**) was attempted (Figure 9B and Supplementary Figure S14). The alkylation efficiently proceeded to the uridine base with a reaction efficiency similar to the thymidine base. Furthermore, we attempted the G-4 RNA alkylation using the human telomere sequence ORN**2** (Supplementary Figure S15). The alkylation also proceeded with a reaction efficiency similar to the G-4 DNA in K^+^ buffer. These RNA alkylation results suggest that the VQ-precursor is a promising reactant for the RNA alkylation with the uridine in the specific hydrophobic pocket as well as for the DNA alkylation.

Next, we examined the alkylation using the aminoacridine-VQ-SPh precursor (**3-SPh**) to the T-T and U-U mismatch, which is the therapeutic target for the myotonic dystrophy type 1 (DM1). It is noted that the target T-T and U-U mismatch sequences consist of two strands of oligonucleotides which were labeled with different fluorophores, i.e. FAM and Alexa647. Thus, the alkylation yield was calculated by combining the yield of each strand. The obtained alkylation yields were then graphed versus the alkylation time (Figure 9C and Supplementary Figure S16). The result clearly showed the effectivity to the T-T mismatch target, while such an effectivity was not followed in the U-U mismatch target, similar to what we observed in our previous result with VDAT (27). This alkylation efficiency of the T-T mismatch was higher than that of VDAT. For the CTG repeat disease DM1, the abnormal expansion of the CTG is 50–2000 repeats. Given that the T-T mismatches are formed in the expanded (CTG)·(CAG) repeats sequence as reported (2,9,42–43), a more efficient alkylation to the long CTG repeats sequence might be possible using the VQ-SPh-conjugated compounds.

## CONCLUSION

**Figure 11.**
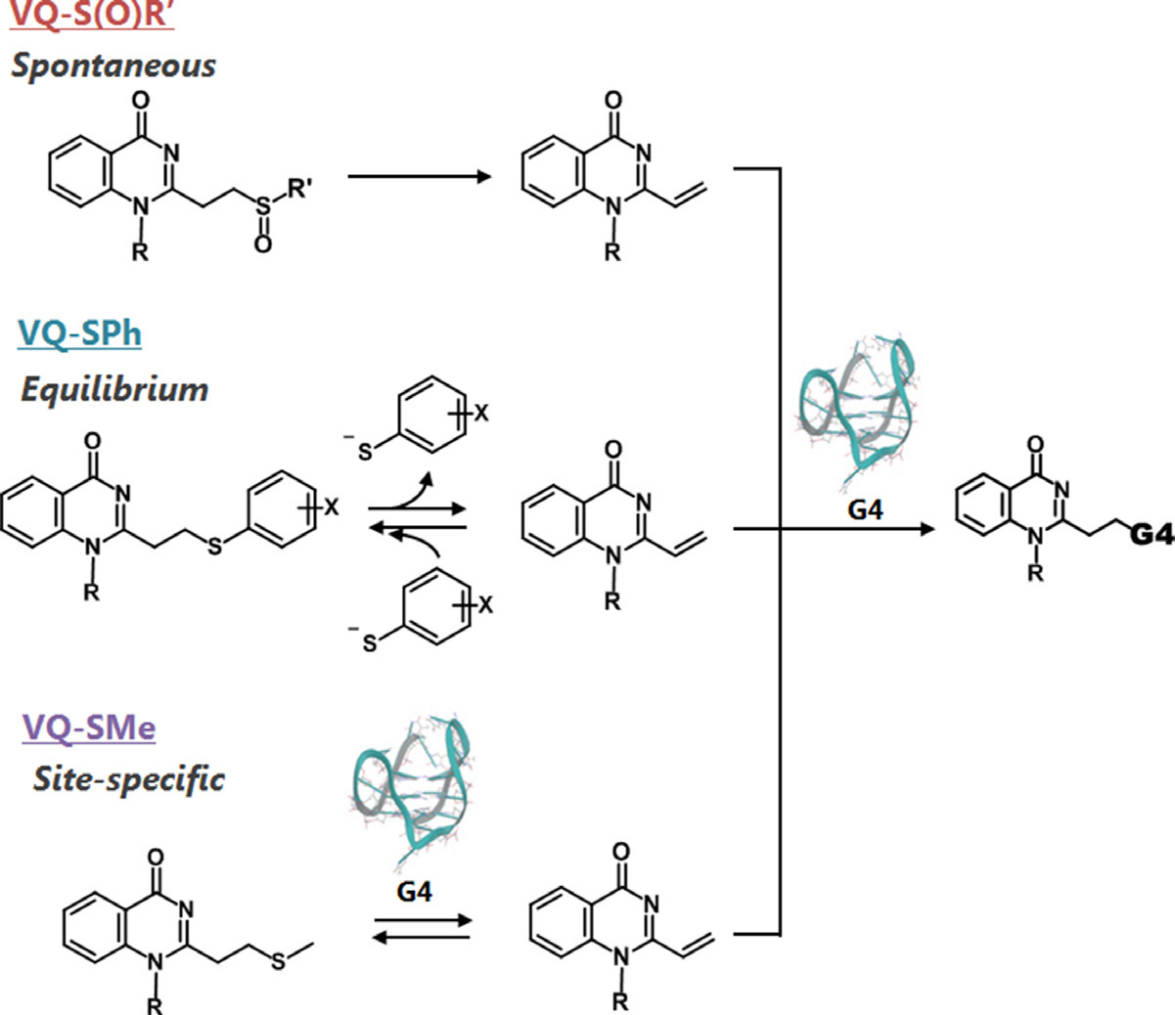
Plausible alkylation mechanism of VQ-S(O)R’ (top), VQ-SPh (middle) and VQ-SMe (bottom) precursors. VQ-S(O)R undergoes spontaneous vinyl generation, VQ-SPh generates vinyl in an equilibrium manner and VQ-SMe site-specifically generates the vinyl.

We attempted to develop reactive OFF-ON type alkylating agents using the aminoacridine-VQ precursors. The VQ-precursors with a sulfoxide, thiophenyl or thiomethyl group, could alkylate the G-quadruplex structure at the *N*3 position of the thymidine base with a significant selectivity over the single- and double-stranded DNAs. Based on a reaction mechanism study, we determined that the VQ in its sulfoxide precursor undergoes a spontaneous vinyl generation, whereas the thiophenyl precursor generates a vinyl in an equilibrium manner, and the sulfide precursor generates the vinyl site-specifically or only when being triggered by the presence of the target DNA site (Figure 11). Based on these results, we can tune the alkylation efficiency and VQ stability by changing the leaving group. We also showed that the VQ-precursor ligand can efficiently alkylate other higher-ordered structures of nucleic acids, e.g. the AP-site (**N** = T, dI or U), G-4 RNA and T-T mismatch. Since the VQ-precursors have an effective reactivity, their conjugates with the DNA or RNA binder are expected to be a potent inhibitor for various targets. For this purpose, chlorambucil derivatives are commonly used and provide a strong inhibition to various targets, such as G-4 DNA (15), repeat RNA (12,14) and pri-miRNA (44), in addition to the duplex DNA (45). The VQ-precursors would also be a candidate for a greater T or U specific alkylation in the higher-ordered structures of DNA or RNA. In addition, the sulfide precursor can site-specifically release the functional group as well as alkylate the target. This unique releasing function might be a potent pro-drug carrier such as click and release strategies (46–49). In this study, the aminoacridine intercalator was utilized as a general binder for the screening of the leaving groups and reactable targets. Based on the positive results of the VQ precursor moiety potential, more selective and efficient alkylations would be possible by means of combining the VQ precursor with the highly selective binders for G-4, T-T mismatched DNA and disease-related RNA.

## Supplementary Material

gkz512_Supplemental_File
