# Supplementary material for: Reactive OFF-ON type alkylating agents for higher-ordered structures of nucleic acids
Source: Nucleic Acids Res. 2019 Jun 12;47(13):6578–89. doi: 10.1093/nar/gkz512 (PMC6649768; doi:10.1093/nar/gkz512)

Supplementary Data

Reactive OFF-ON type alkylating agents for higher-ordered structures of nucleic acids

Kazumitsu Onizuka [1,2,†], Madoka E. Hazemi [1,2,†], Norihiro Sato [1,2], Gen-ichiro Tsuji [1,2], Shunya Ishikawa [1,2], Mamiko Ozawa [1], Kousuke Tanno [1,2], Ken Yamada [1,2] and Fumi Nagatsugi [1,2,*]

[1] Institute of Multidisciplinary Research for Advanced Materials, Tohoku University, 2-1-1 Katahira, Aoba-ku, Sendai, Miyagi 980-8577, Japan

[2] Department of Chemistry, Graduate School of Science, Tohoku University, Aoba-ku, Sendai 980-8578, Japan

Contents

Experimental procedures	S2-S6
Figure S1. Synthesis of VQ precursors	S7
Figure S2. Screening of the leaving group for alkylation	S8
Figure S3. CD spectra of G-4 DNA with **3-SMe**.	S9
Figure S4. Enzymatic hydrolysis of alkylated oligonucleotides	S9
Figure S5. Monomer reaction	S10
Figure S6. Gel electrophoresis of primer extension assay	S11
Figure S7. Reaction of aminoacridine-VQ sulfoxide precursors with glutathione	S12
Table S1. Synthesis of VQ-SPhX precursors and ESI-HRMS measurements	S13
Figure S8. Alkylation reaction of G-4 with VQ-SPhX.	S14
Figure S9. Time course of G-4 alkylation with VQ-SPhX	S15
Figure S10-11. Rerationship between Hammett constant and alkylation yield	S15
Figure S12. Detection of thiophenol by BES-Thio assay	S16
Figure S13. Alkylation by aminoacridine-VQ thiomethyl precursor	S17
Figure S14. Alkylation of AP-site DNA	S18
Figure S15. G-4 RNA alkylation	S18
Figure S16. Alkylation of T-T and U-U mismatch	S19
NMR data	S20-S26

<u>Experimental procedures</u>

Synthesis of *tert*-butyl 2-(2-(2-(methylthio)ethyl)-4-oxoquinazolin-1(4*H*)-yl)acetate (6)

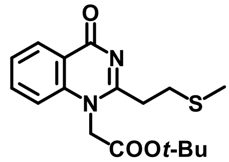

To a solution of 2-aminobenzamide (301 mg, 2.21 mmol) in DMF (4.0 mL), were added K_2CO_3 (919 mg, 6.65 mmol) and *tert*-butyl bromoacetate (485 µL, 3.31 mmol) and stirred at 90 °C. After stirred for 40 h, the mixture was cooled to room temperature and diluted with CH_2Cl_2 (30 mL) and water (10 mL). The organic layer was separated, dried over anhydrous Na_2SO_4, filtered, and evaporated under reduced pressure. The residue was purified by column chromatography ($CHCl_3$/ MeOH = 99/ 1) to give the compound **5** (265.7 mg, 48%) as a pale yellow solid.

To a solution of **5** (100.3 mg, 0.40 mmol) in CH_2Cl_2 (3.5 mL), was added 3-(methylthio)propionyl chloride (140 µL, 1.21 mmol) and stirred at room temperature. After stirred for 3 h, the reaction mixture was diluted with CH_2Cl_2 (10 mL), and washed with saturated aqueous $NaHCO_3$ (15 mL × 4), water (15 mL) and brine (15 mL). The organic layer was dried over anhydrous Na_2SO_4, filtered, and concentrated reduced pressure. The crude was suspended in Et_2O/ hexane = 1/ 2 (10 mL), and the solid was filtered off, followed by washing with Et_2O/ hexane = 1/ 2 (20 mL) to afford the desired product **6** (97.1 mg, 73%) as a pale yellow solid.

^{1}H NMR ($CDCl_3$, 400 MHz) δ (ppm) 8.36 (1H, d, J = 8.0 Hz), 7.74 (1H, dd, J = 7.6, 8.0 Hz), 7.50 (1H, t, J = 7.6 Hz), 7.22 (1H, d, J = 8.8 Hz), 4.86 (2H, brs), 3.14-3.18 (2H, m), 3.07-3.11 (2H, m), 2.30 (2H, brs), 2.18 (3H, s), 1.48 (9H, s). ^{13}C NMR ($CDCl_3$, 125 MHz) δ (ppm) 167.4, 165.6, 162.4, 140.7, 134.1, 128.8, 126.2, 119.8, 114.0, 84.4, 49.0, 35.0, 30.9, 27.9, 15.9. ESI-HRMS (*m*/*z*): $[M+H]^+$ calcd for $C_{17}H_{23}N_2O_3S^+$, 335.1424, found 335.1420.

Synthesis of 2-(2-(2-(methylthio)ethyl)-4-oxoquinazolin-1 (4*H*)-yl)acetic acid (7)

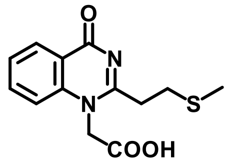

To a solution of compound **6** (41 mg, 0.13 mmol) in DCM (0.2 mL), were added triisopropyl silane (40 µL, 0.19 mmol) and TFA (0.82 mL), then the reaction mixture was stirred at room temperature. After stirred for 4 h, the reaction mixture was then concentrated under reduced pressure and co-evaporated with acetonitrile three times. The residue was purified by column chromatography (EtOAc only → EtOAc: MeOH = 4: 1) to afford compound **7** as a white solid (25 mg, 72%).

^{1}H NMR (DMSO-d_6, 400 MHz) δ (ppm) 8.14 (1H, d, J = 7.6 Hz), 7.87 (1H, dd, J = 7.2, 8.0 Hz), 7.64 (1H, d, J = 8.4 Hz), 7.57 (1H, dd, J = 7.2, 7.6 Hz), 5.24 (2H, s), 3.16 (2H, brs), 2.87 (2H, t, J = 7.2 Hz), 2.49 (2H, br), 2.12 (3H, s). ^{13}C NMR (DMSO-d_6, 125 MHz) δ (ppm) 169.0, 164.4, 163.4, 140.7, 135.3, 127.7, 127.2, 119.6, 116.8, 49.0, 34.0, 30.2, 15.1; ESI-HRMS (*m*/*z*): $[M+H]^+$ calcd for $C_{13}H_{15}N_2O_3S^+$, 279.0798, found 279.0795.

Synthesis of *tert*-butyl(2-(2-(2-(acridin-9-ylamino)ethoxy)ethoxy) ethyl) carbamate (10)

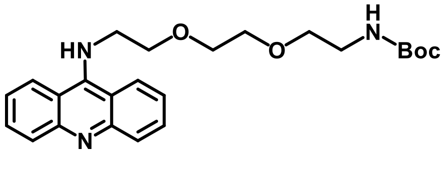

The compound 9-chloroacridine (**8**) (230 mg, 1.08 mmol) and amine linker (**9**) (321 mg, 1.29 mmol) were dissolved in phenol (1.1 g) then the reaction mixture was stirred at 100°C for 3h. The reaction mixture was cooled down to room temperature and was poured 1 N aqueous NaOH (10 mL). The solution was extracted with CH_2Cl_2 (30 mL × 2), washed with brine (20 mL) and dried over anhydrous Na_2SO_4, filtered and evaporated. The residue was purified by column chromatography ($CHCl_3$: MeOH = 9: 1 → 7: 1 → 5: 1 → 3: 1) to afford compound **10** as a yellow oil (442 mg, 96%).
^{1}H NMR ($CDCl_3$, 400 MHz) δ (ppm) 8.18 (2H, d, *J* = 8.8 Hz), 8.11 (2H, d, *J* = 8.8 Hz), 7.66-7.70 (2H, m), 7.39 (2H, dd, *J* = 7.2, 7.6 Hz), 5.00 (1H, br-s), 3.99 (2H, dd, *J* = 4.4, 4.8Hz), 3.68 (4H, br), 3.58-3.62 (2H, m), 3.49 (2H, br), 3.36 (2H, d, *J* = 4.8 Hz), 1.39 (9H, s). ^{13}C NMR ($CDCl_3$, 125 MHz) δ (ppm) 155.84, 151.77, 148.42, 130.05, 128.55, 123.17, 122.96, 117.25, 79.11, 77.26, 70.17, 70.12, 70.04, 49.86, 40.21, 28.23. ESI-HRMS (*m*/*z*): $[M+H]^+$ calcd. for $C_{42}H_{32}N_3O_4^+$, 426.2387; found 426.2396.

Synthesis of *N*-(2-(2-(2-(acridin-9-ylamino)ethoxy)ethoxy)ethyl)-2-(2-(2-(methylthio)ethyl)-4-oxoquinazolin-1(4*H*)-yl)acetamide (3-SMe)

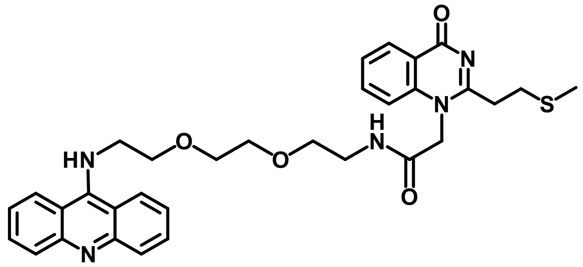

To a solution of compound **10** (14 mg, 0.03 mmol) in DCM (0.2 mL) was added TFA (0.95 mL) then the reaction mixture was stirred at room temperature for 2h. The reaction mixture was then concentrated and co-evaporated with acetonitrile three times. The residue was passed through amino silica, concentrated, and then dissolved in DMF (0.5 mL). The reaction solution was added to a new flask having compound **7** (11 mg, 0.04 mmol) in DMF (0.1 mL). To the reaction mixture were added HBTU (15 mg, 0.04 mmol), HOBt (5.3 mg, 0.04 mmol), DIPEA (58 µL, 0.33 mmol) and the reaction mixture was stirred at room temperature. After stirred for 2 h, the reaction mixture was diluted with DCM, and then washed with sat. aq. $NaHCO_3$ and brine. The organic layer was separated and dried over Na_2SO_4, filtered, and evaporated. The residue was purified by column chromatography (EtOAc : MeOH = 49:1 → 29:1 → 19:1 → 9:1) to afford compound **3-SMe** as a yellow solid (10 mg, 52%). A part of this solid was further purified by reverse phased HPLC with C-18 column (Nacalai tesque: COSMOSIL $5C_{18}$-AR-II, 10×250 mm) by a liner gradient of 0-45%/30min acetonitrile in 0.1% TFA buffer at a flow rate of 4 mL/min at 40°C, and monitored by UV detection at λ = 254 nm and fluorescence detection (λ_{ex} = 266 nm, λ_{em} = 450 nm), to afford the desired product as a pale yellow solid. The concentration of **3-SMe** was determined by quantitative ^{1}H-NMR using maleic acid as an internal standard (ε_{260} = 48,750 $M^{-1}\cdot cm^{-1}$).
^{1}H NMR ((DMSO-d_6, 600 MHz) δ (ppm) 13.48 (1H, s), 9.64 (1H, dd, *J* = 5.4, 6.0 Hz), 8.59 (2H, d, *J* = 9.0 Hz), 8.56 (1H, dd, *J* = 5.4, 6.0 Hz), 8.04 (1H, dd, *J* = 1.2, 7.8 Hz), 7.98 (2H, dd, *J* = 1.2, 8.4 Hz), 7.83 (2H, dd, *J* = 1.2, 8.4 Hz), 7.72 (1H, dd, *J* = 1.2, 8.4 Hz), 7.55 (2H, dd, *J* = 7.2, 7.8 Hz), 7.41 (2H, dd, *J* = 7.2, 8.4 Hz), 4.92 (2H, s), 4.27 (2H, q, *J* = 5.4 Hz), 3.92 (2H, t, *J* = 5.4 Hz), 3.57-3.58 (2H, m), 3.47-3.50 (2H, m), 3.36 (2H, t, *J* = 5.4 Hz), 3.19 (2H, dd, *J* = 5.4, 11.4 Hz), 3.04 (2H, br-s), 2.85 (2H, t, *J* = 7.8 Hz), 2.09 (3H, s). ^{13}C NMR ((DMSO-d_6, 150 MHz) δ (ppm) 167.2, 166.2, 163.1, 158.3, 158.1, 157.8, 141.2, 135.3, 133.9, 127.3, 125.6, 123.4, 119.3, 118.6, 115.5, 69.9, 69.4, 68.8, 68.2, 49.0, 48.7, 40.1, 38.8, 34.3, 29.9, 14.9. ESI-HRMS (*m*/*z*): $[M+H]^+$ calcd for $C_{32}H_{36}N_5O_4S^+$, 586.2483; found 586.2484.

Synthesis of aminoacridine-VQ conjugate methylsulfoxide precursor (3-S(O)Me)

To a solution of **3-SMe** (5 nmol) in DMSO (0.5 µL) was added a solution of MMPP (5 nmol) in water (9.5 µL) and the mixture was rest at room temperature for 1 min to afford compound **3-S(O)Me**. The precursor was used for alkylation reactions without further purification. For ESI-MS and NMR measurements, the **3-S(O)Me** was purified by reverse phased HPLC with C-18 column (Nacalai tesque: COSMOSIL $5C_{18}$-AR-II, 10×250 mm) by a liner gradient of 0-45%/30min acetonitrile in 0.1% TFA buffer at a flow rate of 4 mL/min at $40^{o}C$, and monitored by UV detection at λ = 254 nm and fluorescence detection (λ_{ex} = 442 nm, λ_{em} = 494 nm). ESI-HRMS (*m*/*z*): $[M+2H]^{2+}$ calcd for $C_{32}H_{37}N_5O_5S^{2+}$, 269.6261; found 269.6273.

Synthesis of aminoacridine-VQ conjugate thiophenol (3-SPh) precursors

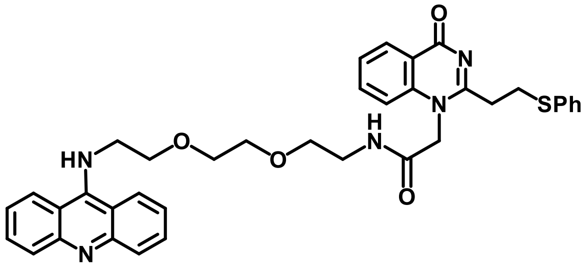

To a solution of **3-SMe** (2 nmol) in DMSO (2 µL) was added a solution of MMPP (1.2 nmol) in water (1.2 µL) and the mixture was rest at room temperature for 1 min to afford compound **3-S(O)Me**. Carbonate buffer pH 10 (50 mM, 0.4 µL) and thiophenol (100 nmol) in DMSO (0.2 µL), and DMSO (1.2 µL) were then added and the mixture was incubated at 37°C for 3 h. The solution was purified by HPLC to afford compound **3-SPh**.

Large scale synthesis: To a solution of **3-SMe** (11.8 µmol) in DMSO (250 µL) and H_2O (930 µL) was added a solution of MMPP (10.8 µmol) in water (708 µL) and the mixture was rest at room temperature for 1 min to afford compound **3-S(O)Me**. Carbonate buffer pH 10 (50 mM, 232 µL), thiophenol (5.9 mmol) in DMSO (116 µL), and DMSO (690 µL) were then added and the mixture was incubated at 37°C for 3 h. To the solution was added 2-2'-dipyridyldisulfide (2.9 mmol) in DMSO (58 µL) then the solution was purified by HPLC to afford compound **3-SPh**.

^{1}H NMR (600 MHz, DMSO-d_6) of **3-SPh**: δ (ppm) = 13.42 (1H, s), 9.60 (1H, t, *J* = 5.4 Hz), 8.59 (2H, d, *J* = 8.4 Hz), 8.48 (1H, t, *J* = 5.4 Hz), 8.05 (1H, dd, *J* = 7.8, 1.8 Hz), 7.97 (2H, dd, *J* = 8.4, 7.2 Hz), 7.82 (2H, d, *J* = 8.4 Hz), 7.71 (1H, ddd, *J* = 7.8, 7.2, 1.8 Hz), 7.54 (2H, t, *J* = 8.4 Hz), 7.43 – 7.39 (2H, m), 7.33 (2H, d, *J* = 7.2 Hz), 7.29 (2H, t, *J* = 7.2 Hz), 7.16 (1H, t, *J* = 7.2 Hz) 4.9 (2H, s), 4.27 (2H, q, *J* = 5.4 Hz), 3.91 (2H, t, *J* = 5.4 Hz), 3.57 (2H, t, *J* = 5.4 Hz), 3.47 – 3.45 (2H, m), 3.36 – 3.31 (4H, m), 3.15 (2H, t, *J* = 5.4 Hz), 3.07 (2H, br). ^{13}C NMR (150 MHz, DMSO-d_6) of **3-SPh**: δ (ppm) = 167.26, 166.07, 162.60, 158.23, 157.70, 141.18, 135.97, 135.21, 133.78, 129.09, 128.11, 127.26, 125.77, 125.50, 119.28, 118.52, 115.23, 69.84, 69.40, 68.73, 68.16, 48.91, 48.64, 38.71, 34.18, 28.97. ESI-HRMS (*m*/*z*): $[M+H]^{+}$ calcd. for $C_{37}H_{38}N_5O_4S^{+}$, 648.2639; found 648.2649.

Synthesis of aminoacridine-VQ conjugate phenylsulfoxide precursor (3-S(O)Ph)

To a solution of **3-SPh** (5 nmol) in DMSO (0.5 µL) was added a solution of MMPP (5 nmol) in water (9.5 µL) and the mixture was rest at room temperature for 1 min to afford compound **3-S(O)Ph**. The precursor was used for alkylation reactions without further purification.

General procedure of synthesis of aminoacridine-VQ conjugate 4-methoxythiophenol precursor (3-SPhX)

To a solution of **3-SMe** (2 nmol) in H_2O (2 µL) was added a solution of MMPP (1.2 nmol) in water (1.2 µL) and the mixture was rest at room temperature for 1 min. Carbonate buffer pH 10 (50 mM, 0.4 µL), thiophenol derivatives (100 nmol) in DMSO (0.2 µL), and DMSO (1.2 µL) were then added

and the mixture was incubated at 37°C or 45°C (Table S1). The solution was purified by HPLC to afford compound **3-SPhX**.

Annealing procedure
DNA and/or RNA (50 µM) were diluted in these following 1x buffers (50 µL).
K^+ buffer: 100 mM KCl, 10 mM K_2HPO_4/KH_2PO_4 (pH 7.0), 1 mM K_2EDTA.
Na^+ buffer: 100 mM NaCl, 10 mM Na_2HPO_4/NaH_2PO_4 (pH 7.0), 1 mM Na_2EDTA.
MES buffer: 100 mM NaCl, 50 mM MES (pH 7.0).
To perform the thermal annealing of DNA and/or RNA, the DNA solution was heated at 90 ^{o}C for 10 min and cooled from 90 ^{o}C to 20 ^{o}C at a rate of 0.5 ^{o}C/min by a thermal cycler.

Alkylation of targets
Alkylation of G-4 DNA in the presence of 2-2'-dipyridyldisulfide
To a solution (10 µL) of VQ-precursor ligand (50 µM), and DNA [G-4] (2.5 µM) in 1× K^+ buffer pH 7.0 (containing 2% DMSO) was added 2,2'-dipyridyldisulfide (50 µM) and the reaction mixture was incubated at 37°C.
Alkylation of G-4 DNA under acidic conditions (pH 6.5)
A solution (10 µL) of VQ-precursor ligand (50 µM), and DNA [G-4] (2.5 µM) in 1× K^+ buffer pH 6.5 (containing 2% DMSO) was incubated at 37°C.

CD measurements
A solution (100 µL) of G4-DNA (2.5 µM) and **3-SMe** (0, 2.5, 12.5 or 50 µM) in 1× K^+ or Na^+ buffer (containing 2% DMSO, pH 7.0) were transferred to a cylindrical micro cell with a 1-cm path length. The measurement was performed at 25 ^{o}C by a J-720WI (JASCO Co., Hachioji, Japan) equipped with a Peltier temperature controller.

Enzymatic hydrolysis of alkylated DNA
A solution (10 µL) of alkylated DNA (100 µM), alkaline phosphatase (0.03 U/µL, Takara Bio Inc.) and phosphodiesterase I (0.03 U/µL, Worthington Biochemical Corp.) in 1× alkaline phosphatase buffer was incubated at 37 ^{o}C for 2 h. The solution mixture was analyzed by RP-HPLC. (HPLC conditions: C-18 column (Shiseido CAPCELL PAK C18-MG-II, 4.6 × 250 mm) by a linear gradient of 5%-40%/30min acetonitrile in 0.1M TEAA at a flow rate of 1 mL/min, at 40 ^{o}C. Each peaks was monitored by UV detector (λ = 254 nm).

Monomer reaction with thymidine
To a solution of **3-SMe** (8.54 µmol) in DMSO (170 µL) and H_2O (684 µL) was added MMPP (5.12 µmol) in H_2O (512 µL). The solution was rest at r.t. for 2 min and then purified by HPLC to afford **3-S(O)Me**. To the purified **3-S(O)Me** was added thymidine (8.54 µmol) in DMSO (854 µL) and the reaction mixture was incubated at 37 ^{o}C for 3 days. The reaction mixture was purified by HPLC to afford thymidine-VQ adduct (**VQ-T****). HPLC conditions: C-18 column (Nacalai tesque: COSMOSIL 5C18-AR-II, 4.6 × 250 mm) by a linear gradient of 0%-45%/30min acetonitrile with 0.1% TFA in H_2O with 0.1% TFA at a flow rate of 1 mL/min at 40 ^{o}C. Peak(s) were monitored by UV detector (λ = 254 nm).

Primer extension assay
G-4 template alkylation: a solution (10 µL) of G-4 template (2.5µM) and compound **3-SPh** (50 µM) in 1× K^+ or Na^+ buffer was incubated at 37 ^{o}C for 24 h.
Primer extension: a mixture of solution (10 µL) of the primer G-4 (0.15 µM) and the alkylated template G-4 (0.5 µM) in NEBuffer 2 (50 mM NaCl, 10 mM Tris-HCl, 10 mM $MgCl_2$, 1 mM DTT, pH 7.9, New England Biolabs) were heated at 93 ^{o}C for 3 min and gradually cooled to room

temperature for annealing. To the solution were added dNTPs (0.8 µL, final 0.2 mM) and the Klenow Fragment (exo-) (0.4 µL, final 0.1 U/µL, New England Biolabs), then the mixture was incubated at 37 ^{o}C for 5 min. The reaction mixture was quenched by loading a buffer (80% formamide, 10 mM EDTA, 10 µL), heated to 93 ^{o}C for 5 min, then rapidly cooled to 0 ^{o}C. PAGE was performed on a 14% denaturing polyacrylamide gel containing 30% formamide in 1× TBE and 5.3 M urea at 300 V and 40 ^{o}C for 90 min.

Measurement of precursor's stability by glutathione (GSH) reaction

A solution (25 µL) of VQ-precursor ligand (50 µM) and glutathione (GSH) (1 mM) in 1× K^+ buffer pH 7.0 (2% DMSO) was divided into 5 microtubes (5 µL) incubated at 37 ^{o}C. The reaction mixture was collected at various point of time and analyzed by HPLC (JASCO HPLC System: PU-2089Plus, UV-2075Plus, FP-2015Plus and CO-2065Plus). HPLC analysis conditions: C-18 column (Nacalai tesque: COSMOSIL 5C18-AR-II, 4.6 x 250 mm) by a linear gradient of 0%-45%/30min acetonitrile with 0.1% TFA in H2O with 0.1% TFA at a flow rate of 1 mL/min at 40 ^{o}C. Peak(s) were monitored by UV detector (λ = 254 nm). The first-order rate constant of alkylation reaction (k_{obs}) was graphically obtained from the first-order kinetic plot (Equation 1). The rate k_{obs} values were determined from at least three separate experiments and the half-time ($t_{1/2}$) was calculated from the obtained k_{obs} value.

$\ln([\text{ligand}]_t / [\text{ligand}]_0) = -k_{obs} \cdot t$ (1)

BES Thio assay

Three separated solution (10 µL) of:

[VQ-precursor + G-4 + BES-Thio]: VQ-precursor ligand (50 µM), G-4 DNA (2.5 µM), BES-Thio (25 µM) in 1× K^+ buffer pH 7.0 (2% DMSO)

[G-4 + BES-Thio]: G-4 DNA (2.5 µM), BES-Thio (25 µM) in 1× K^+ buffer pH 7.0 (2% DMSO)

[BES-Thio]: BES-Thio (25 µM) in 1× K^+ buffer pH 7.0 (2% DMSO)

were incubated at 37°C. Aliquots (10 µL) of the reaction mixture were collected at various point of time and diluted with water (60 µL). The solution was transferred to a microquartz cell with a 1-cm path length and analyzed by fluorimeter. The excitation wavelength was set to 495 nm and the emission was recorded from 510 nm to 600 nm at 25 ^{o}C. Fluorescence measurements were performed with a JASCO-6500 spectrofluorometer (JASCO, Tokyo, Japan).

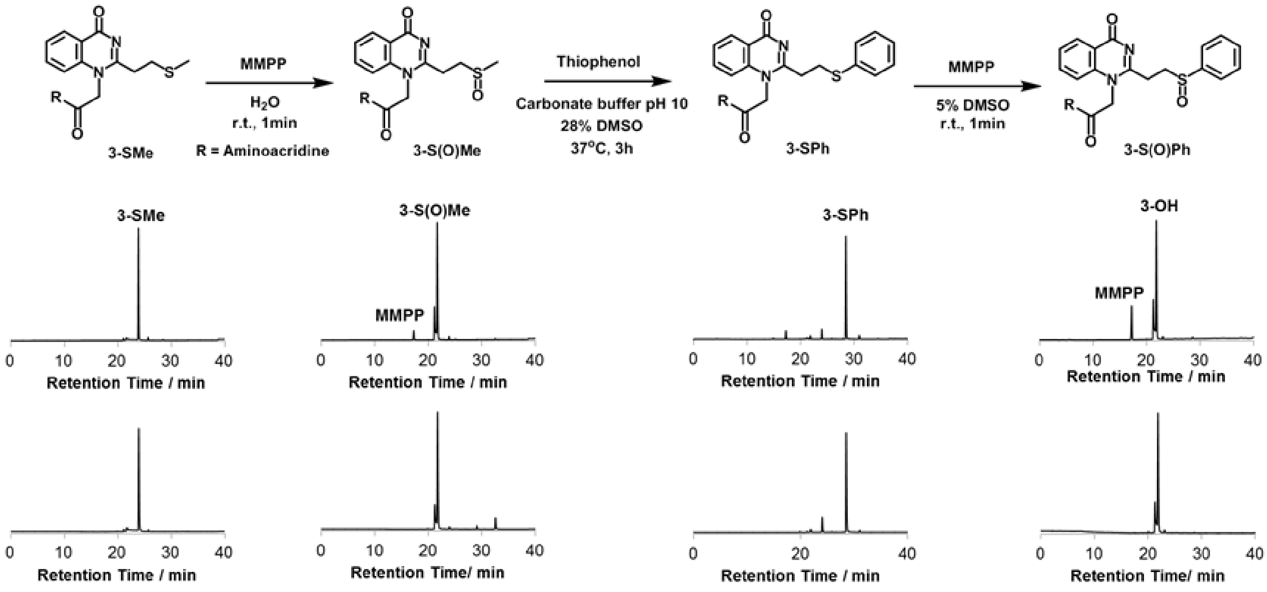

[M+H]$^{+}$:
Calcd.: 586.2483
Found: 586.2486

[M+H]$^{+}$:
Calcd.: 602.2432
Found: 602.2426
[compound**2**+H]$^{+}$:
Calcd.: 538.2449
Found: 538.2449
[**3-OH**+H]$^{+}$:
Calcd.: 556.2554
Found: 556.2555

[M+H]$^{+}$:
Calcd.: 324.6356
Found: 324.6372

[**3-OH**+2H]$^{2+}$:
Calcd.: 278.6314
Found: 278.6321

Figure S1. Synthesis of VQ precursors. HPLC profiles of aminoacridine-VQ conjugate **3-SMe** conversions, confirmed by ESI-MS. HPLC analysis conditions: C-18 column (Nacalai tesque: COSMOSIL 5C18-AR-II, 4.6 x 250 mm) by a linear gradient of 0%-45%/30min ACN with 0.1% TFA in H_2O with 0.1% TFA at a flow rate of 1 mL/min at 40°C. Peaks were monitored by UV-detector (λ=254 nm) (top); FP-detector (λ_{ex}=442 nm; λ_{em}=494 nm) (bottom).

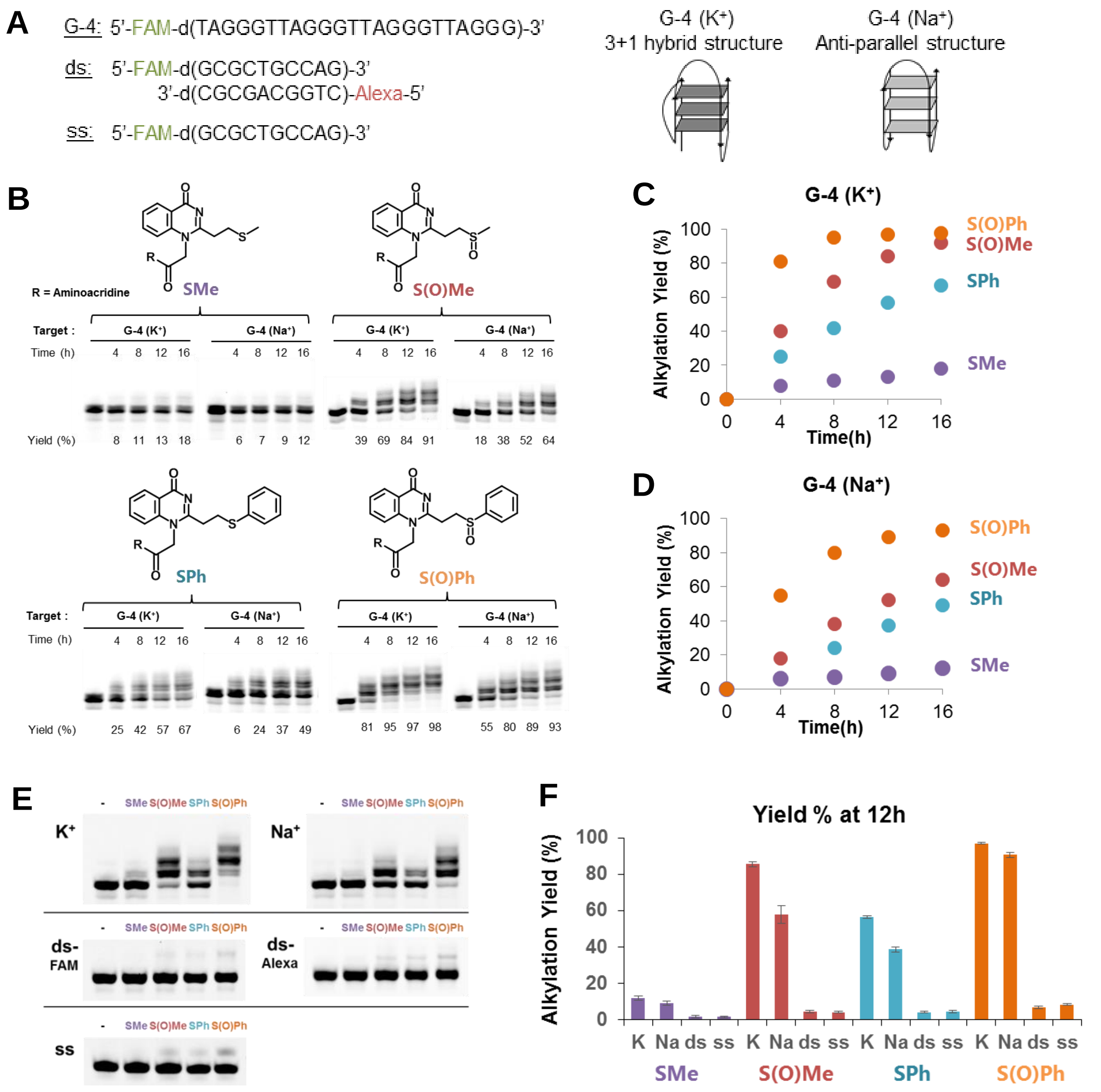

Figure S2. Screening of the leaving group for alkylation. The precursor ligand (50 μM) was incubated with G-quadruplex (G-4) (2.5 μM), duplex (ds) (2.5 μM), and single-stranded (ss) (2.5 μM) DNA at 37°C in buffer solution. G-4 potassium buffer (K^+): 100 mM KCl, 10 mM K_2HPO_4/KH_2PO_4 (pH 7.0), 1 mM K_2EDTA, G-4 sodium buffer (Na^+): 100 mM NaCl, 10 mM Na_2HPO_4/NaH_2PO_4 (pH 7.0), 1 mM Na_2EDTA, ds and ss buffer: G-4 sodium buffer. (A) The target sequences: G-quadruplex (G-4), duplex (ds) and single-stranded (ss) DNA. G-4 (K^+) and G-4 (Na^+) form 3+1 hybrid and anti-parallel structure, respectively. (B) Denaturing gel electrophoresis of the alkylation products for G-4. The electrophoresis was performed on a 16% denaturing polyacrylamide gel containing 20% formamide. (C) Time course of the reaction yields in G-4 potassium buffer (K^+). (D) Time course of the reaction yields in G-4 sodium buffer (Na^+). (E) Denaturing gel electrophoresis of the alkylation products for G-4, ds or ss after 12 h. (F) Bar graph of yield (%) at 12 h (n = 3, error bars indicate standard deviation).

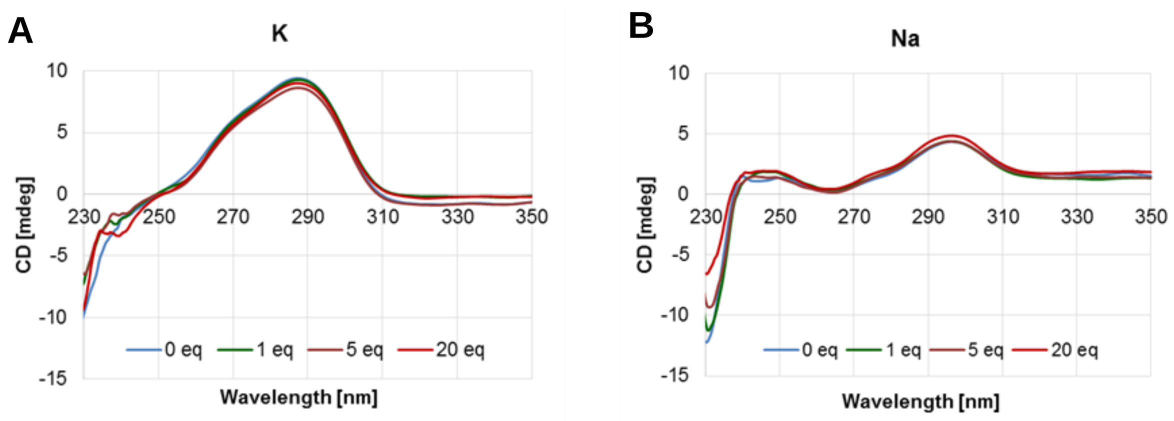

Figure S3. CD spectra of G-4 DNA with **3-SMe**. (A) Potassium buffer (K^+) (B) Sodium buffer (Na^+). CD spectra were measured using G4-DNA (2.5 μM) and **3-SMe** (0, 2.5, 12.5 or 50 μM) in 1× K^+ or Na^+ buffer (containing 2% DMSO, pH 7.0) at 25 °C.

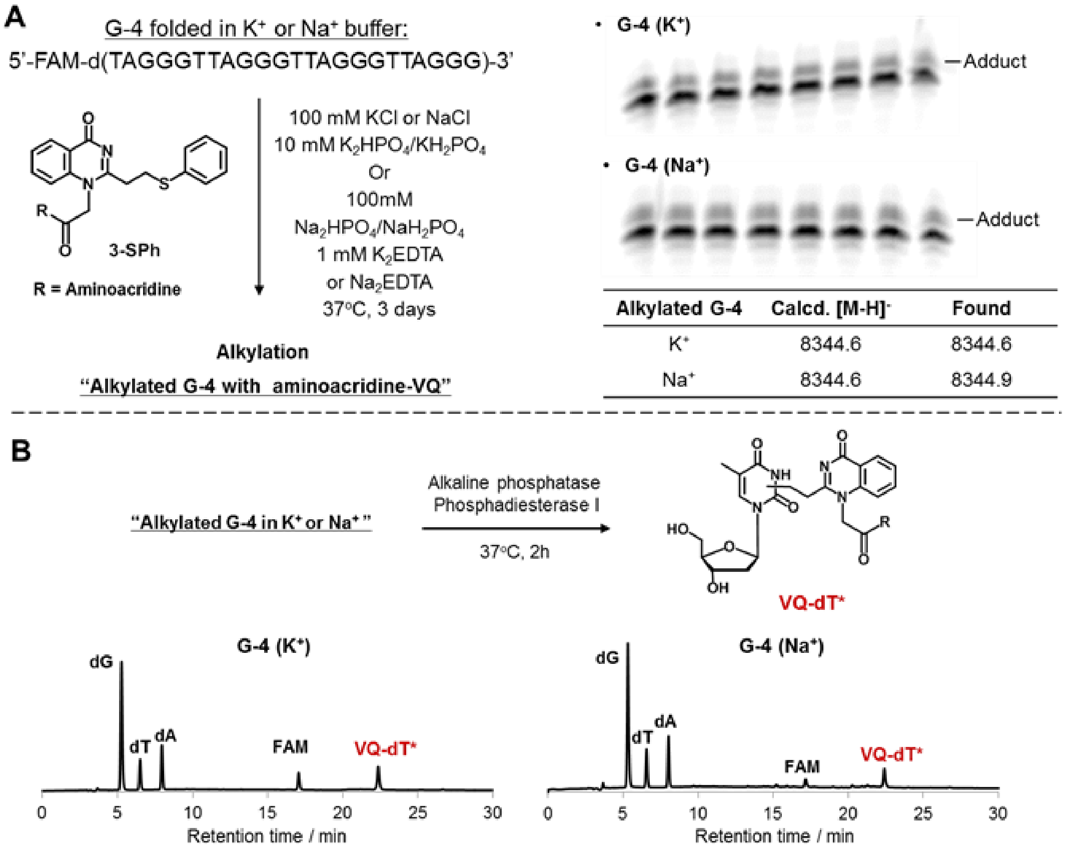

Figure S4. Enzymatic hydrolysis of alkylated oligonucleotides. (A) Synthesis and purification of the alkylated FAM labelled G-4 DNA using VQ-thiophenol precursor (**3-SPh**). The purification of the alkylated G-4 DNA (G-4 adduct) was performed by PAGE. The alkylated DNA product was confirmed by MALDI-TOF mass spectroscopy measurement. (B) Enzymatic hydrolysis of alkylated oligonucleotides. The enzymatic hydrolysis was performed with alkylated G-4 (100 μM), alkaline phosphatase (0.03 U/ μl) and phosphodiesterase I (0.03 U/ μl). After enzymatic hydrolysis, the hydrolyzates were analyzed by HPLC. Alkylated G-4 DNA in K^+ buffer (left) and Na^+ buffer (right). The peak eluting at 24 min was corresponded as VQ-dT* determined by ESI-MS. HPLC analysis conditions: C-18 column (CAPCELL PAK C18 MG-II, 4.0 x 250 mm) by a linear gradient of 5%-40%/30 min of 100 mM TEAA buffer with acetonitrile at a flow rate of 1 mL/min at 40°C. Peaks were monitored by UV detector (λ=254 nm).

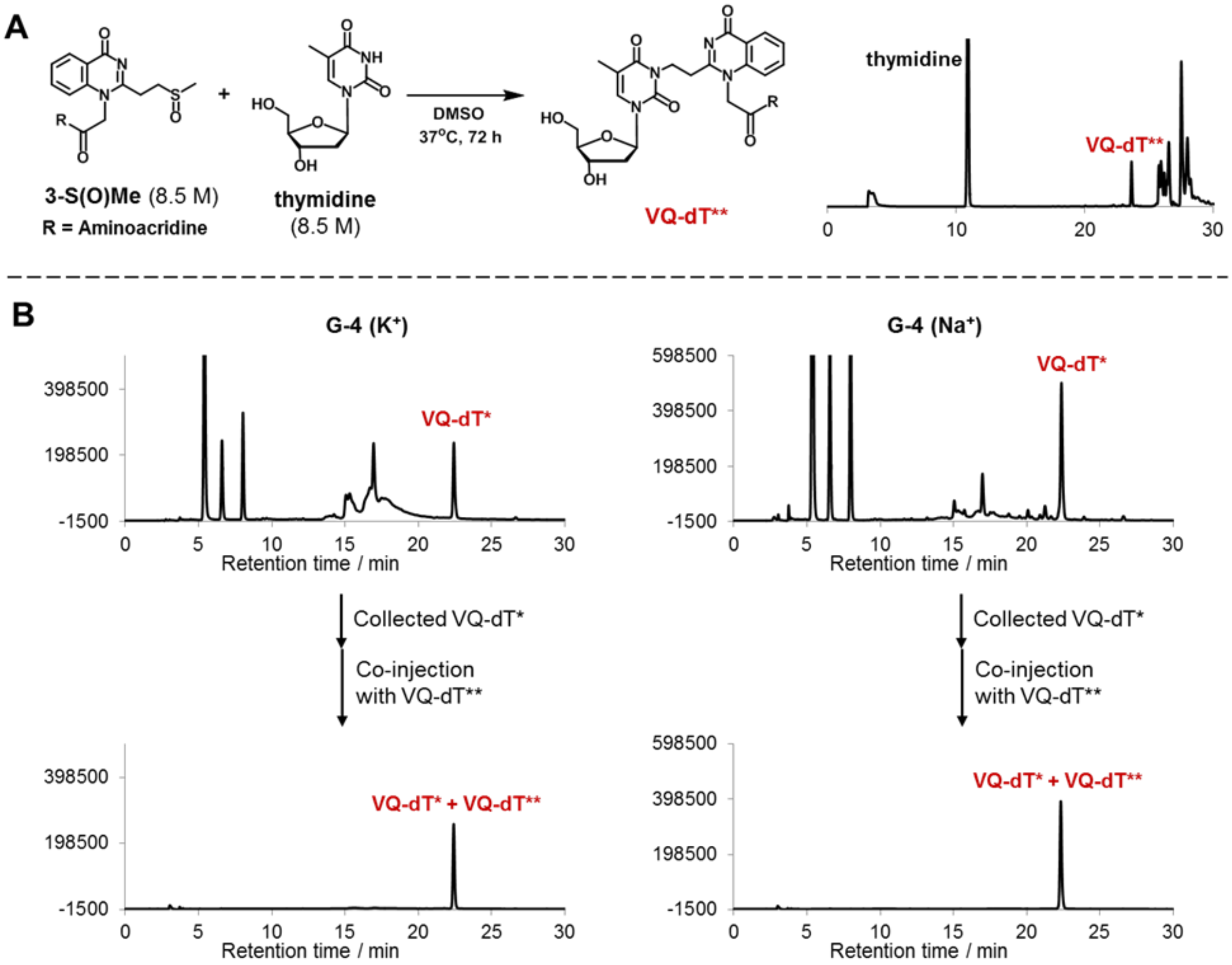

Figure S5. Monomer reaction and co-injection of nucleoside adducts VQ-dT* and VQ-dT**. (A) Monomer reaction between VQ-methylsulfoxide precursor (**3-S(O)Me**) and thymidine. **3-S(O)Me** (8.5 M) was incubated with thymidine (8.5 M) in DMSO for 72h at 37°C. The monomer reaction was analyzed by HPLC. The peak eluting at 11 min was corresponded to starting material thymidine. The peak eluting at 24 min was corresponded as VQ-dT** determined by ESI-MS. HPLC analysis conditions: C-18 column (Nacalai tesque: COSMOSIL 5C18-AR-II, 4.6 x 250 mm) by a linear gradient of 0%-45%/30 min acetonitrile with 0.1% TFA in H2O with 0.1% TFA at a flow rate of 1 mL/min at 40°C. Peak(s) were monitored by UV detector (λ=254 nm). (B) Co-injection of nucleoside adducts VQ-dT* from enzyme hydrolysis of alkylated oligonucleotide and VQ-dT** from monomer reaction. K^+ buffer (left) and Na^+ buffer (right) products. HPLC analysis conditions: C-18 column (CAPCELL PAK C18 MG-II, 4.0 x 250 mm) by a linear gradient of 5%-40%/30min of 100 mM TEAA buffer with acetonitrile at a flow rate of 1 mL/min at 40 ^{o}C. Peak(s) were monitored by UV detector (λ=254 nm).

G-4 Template (ODN**4**):

23 17 11 9

5’-d(TAG GGT TAG GGT TAG GGT TAG GGC AGA GAG)-3’

Primer-11mer (ODN**6**): 3’-d(TC CCG TCT CTC) FAM-5’

Control-17mer: 3’-d(TC CCA ATC CCG TCT CTC) FAM-5’

Control-23mer: 3’-d(TC CCA ATC CCA ATC CCG TCT CTC) FAM-5’

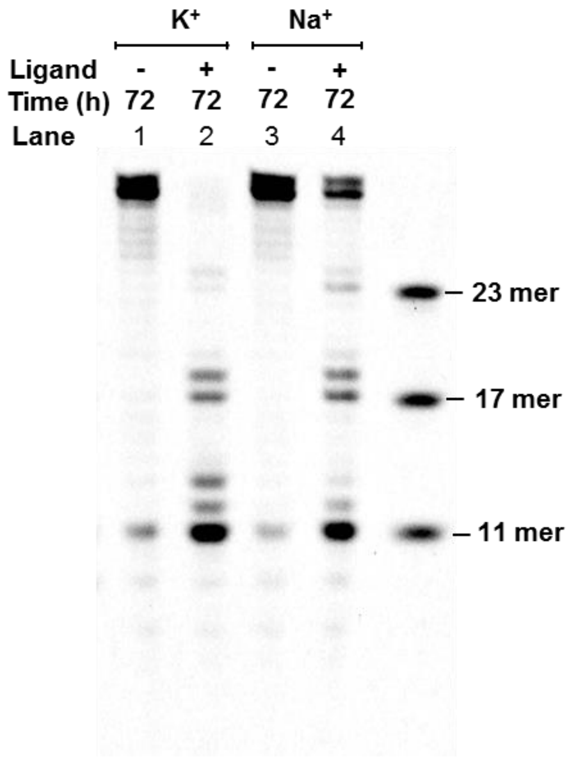

Figure S6. Gel electrophoresis of primer extension assay of non-alkylated and alkylated G-4 DNA. Alkylation was carried out with the G-4-template ODN**4** (2.5 µM) and compound **3-SPh** (50 µM) in K^+ buffer or Na^+ buffer for 72 h. The alkylated template DNA was then annealed with primer-11 (ODN**6**). The primer extension reactions were performed by incubating the G-4 template (5 µM) and primer DNA (0.15 µM), Klenow Fragment (exo$^-$) (0.1 U/µL) and dNTP (0.2 mM) in NE buffer pH 7.9 at 37°C for 5 min.

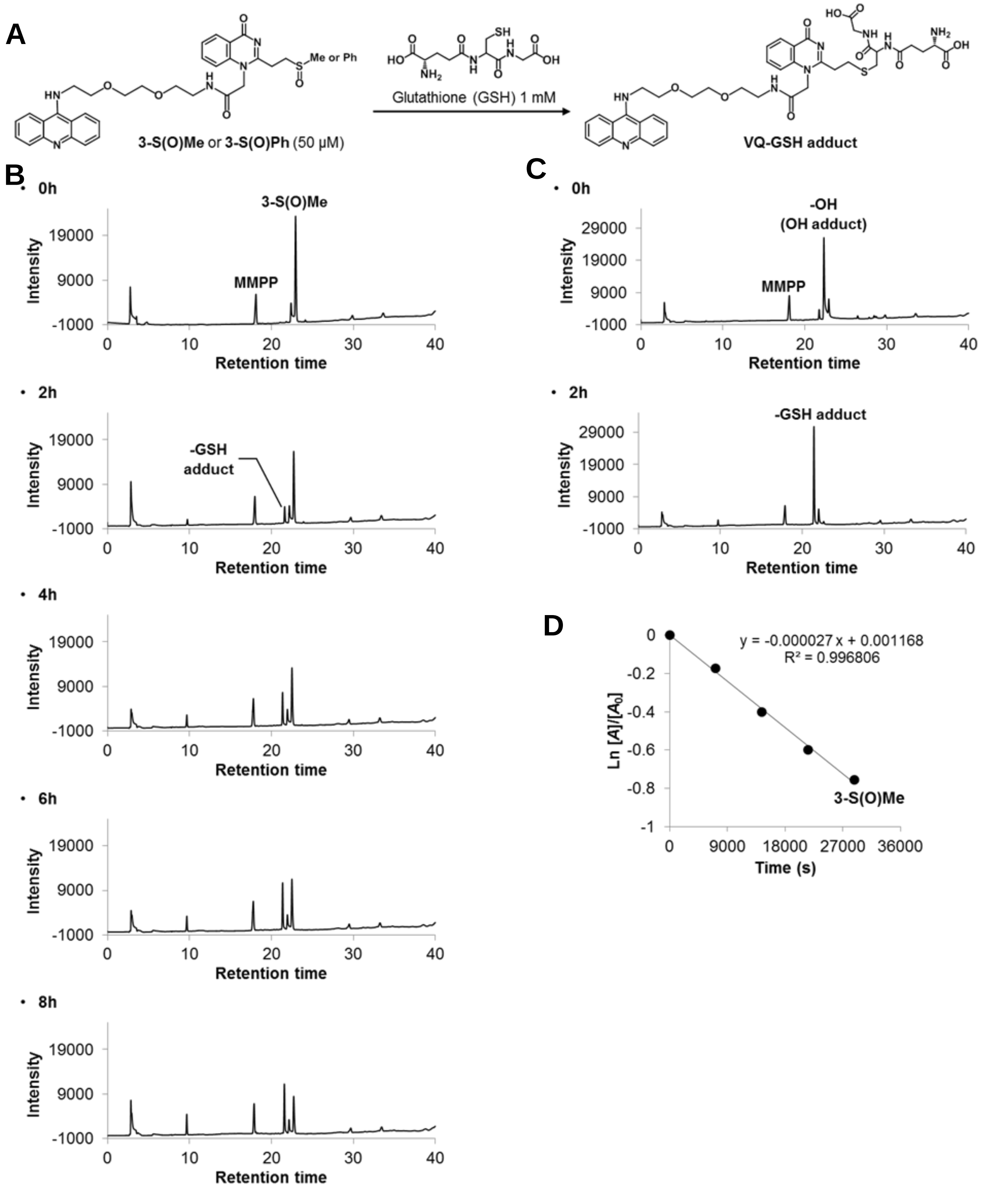

Figure S7. Reaction of aminoacridine-VQ sulfoxide precursors with glutathione (GSH). (A) The precursor ligand (50 μM) was incubated with glutathione (GSH) (1 mM) in G4 potassium buffer (K^+): 100 mM KCl, 10 mM K_2HPO_4/KH_2PO_4 (pH 7.0), 1 mM K_2EDTA, at 37 °C. (B) HPLC analysis of GSH reaction with VQ-methylsulfoxide precursor (**3-S(O)Me**) at 0, 2, 4, 6 and 8 h (top to bottom). GSH adduct was eluted at around 21 min retention time. (C) HPLC analysis of GSH reaction with VQ-phenylsulfoxide precursor (**3-S(O)Ph**) at 0 and 2h (top to bottom). **3-S(O)Ph** was hydrolysed through HPLC analysis giving hydrolysed product eluted at 22 min. GSH adduct was eluted at around 21 min retention time. (D) Calculation of reaction rate for **3-S(O)Me**. HPLC analysis conditions: C-18 column (Nacalai tesque: COSMOSIL 5C18-AR-II, 4.6 x 250 mm) by a linear gradient of 0%-45%/30 min acetonitrile with 0.1% TFA in H_2O with 0.1% TFA at a flow rate of 1 mL/min at 40 °C. Peak(s) were monitored by UV detector (λ=254 nm).

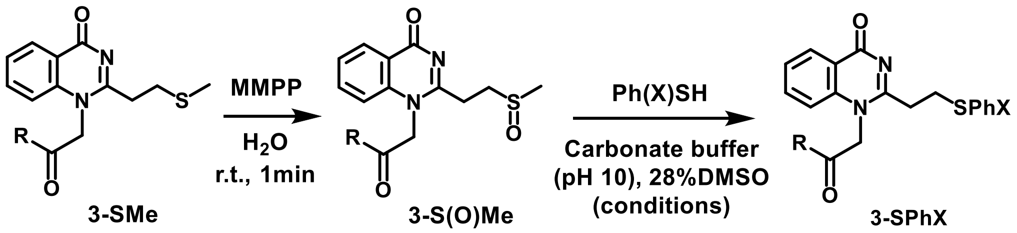

Table S1. Synthesis of VQ-SPhX precursors and ESI-HRMS measurements.

Entry	X =	Conditions	Calcd.	Found
1	*p*-OMe	37 °C, 3 h	678.2745	678.2745
2	*p*-Me	37 °C, 3 h	662.2796	662.2796
3	*m*-Me	37 °C, 3 h	662.2796	662.2797
4	*p*-F	37 °C, 5 h	666.2545	666.2555
5	*m*-OMe	37 °C, 3 h	678.2745	678.2747
6	*p*-Cl	37 °C, 5 h	682.2249	682.2243
7	*m*-F	45 °C, 4 h	669.2545	669.2556

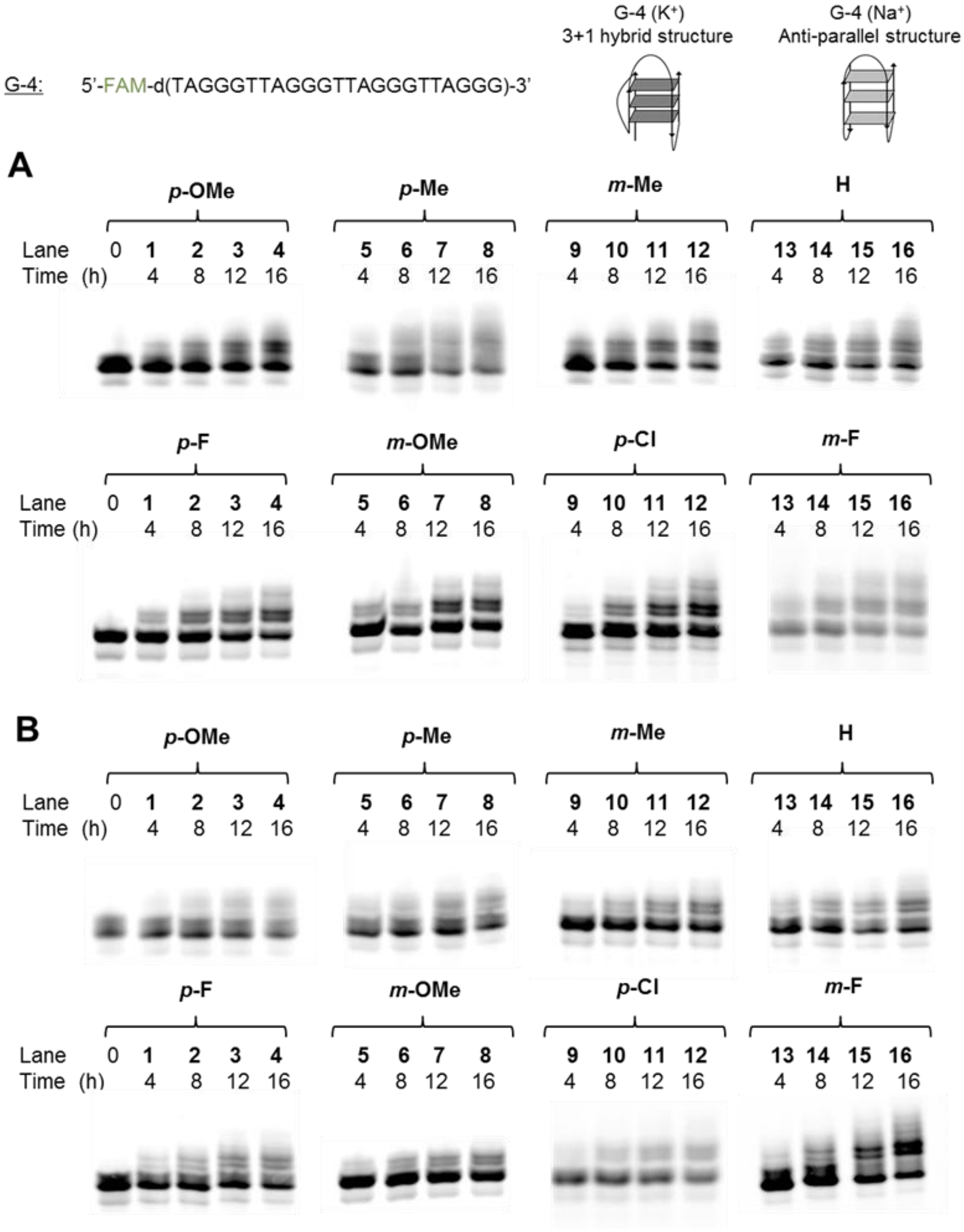

Figure S8. Alkylation reaction of G-4 with VQ-SPhX. The precursor ligand (50 μM) was incubated with G-quadruplex (G-4) DNA folded in K^+ or Na^+ (2.5 μM) at 37°C in buffer solution. G-4 potassium buffer (K^+): 100 mM KCl, 10 mM K_2HPO_4/KH_2PO_4 (pH 7.0), 1 mM K_2EDTA; G-4 sodium buffer (Na^+): 100 mM NaCl, 10 mM Na_2HPO_4/NaH_2PO_4 (pH 7.0), 1 mM Na_2EDTA. (**A**) Denaturing gel electrophoresis of the alkylation products for G-4 in K^+ buffer. Lane 0 is the control G-4 DNA without ligand. (**B**) Denaturing gel electrophoresis of the alkylation products for G-4 in Na^+ buffer. The electrophoresis was performed on a 16% denaturing polyacrylamide gel with 20% formamide.

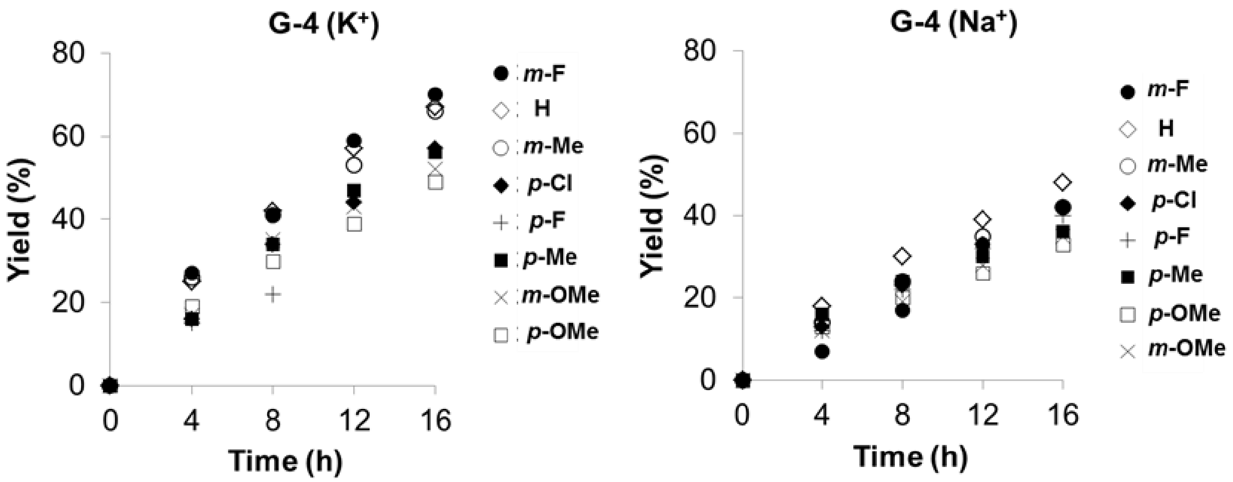

Figure S9. Time course of G-4 alkylation with VQ-SPhX in K^+ (left) and Na^+ (right) buffers.

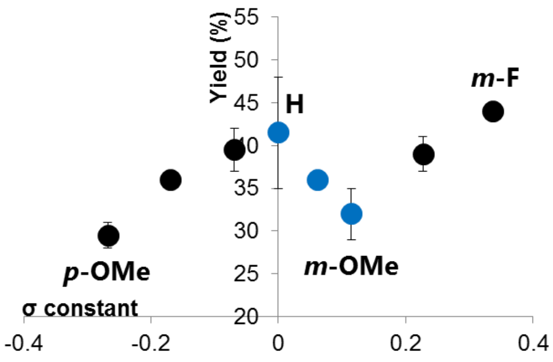

Figure S10. Rerationship between Hammett constant and alkylation yield in Na^+ buffer for VQ-SPhX precursors (n = 2, error bars indicate standard deviation).

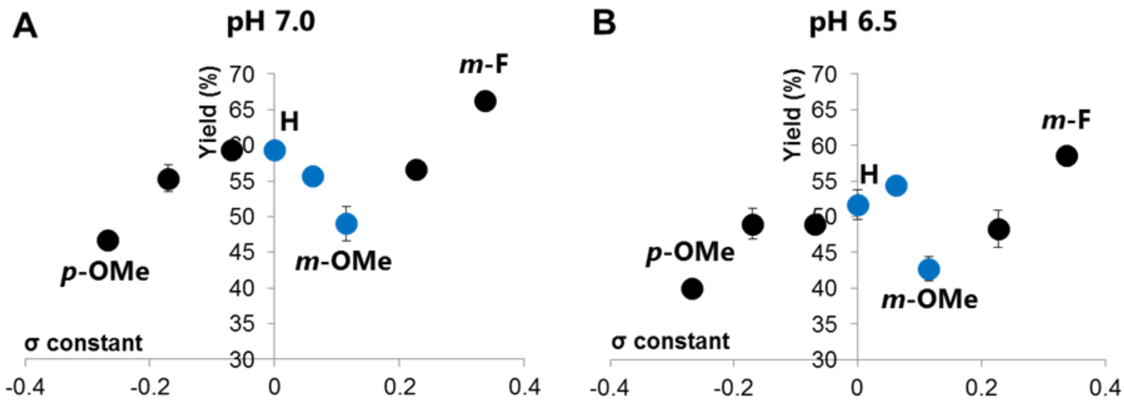

Figure S11. Rerationship between Hammett constant and alkylation yield for VQ-SPhX precursors. (**A**) The reaction with G-4 in K^+ buffer at pH 7.0 (n = 3, error bars indicate standard deviation). (**B**) Reaction at pH 6.5 (n = 3, error bars indicate standard deviation).

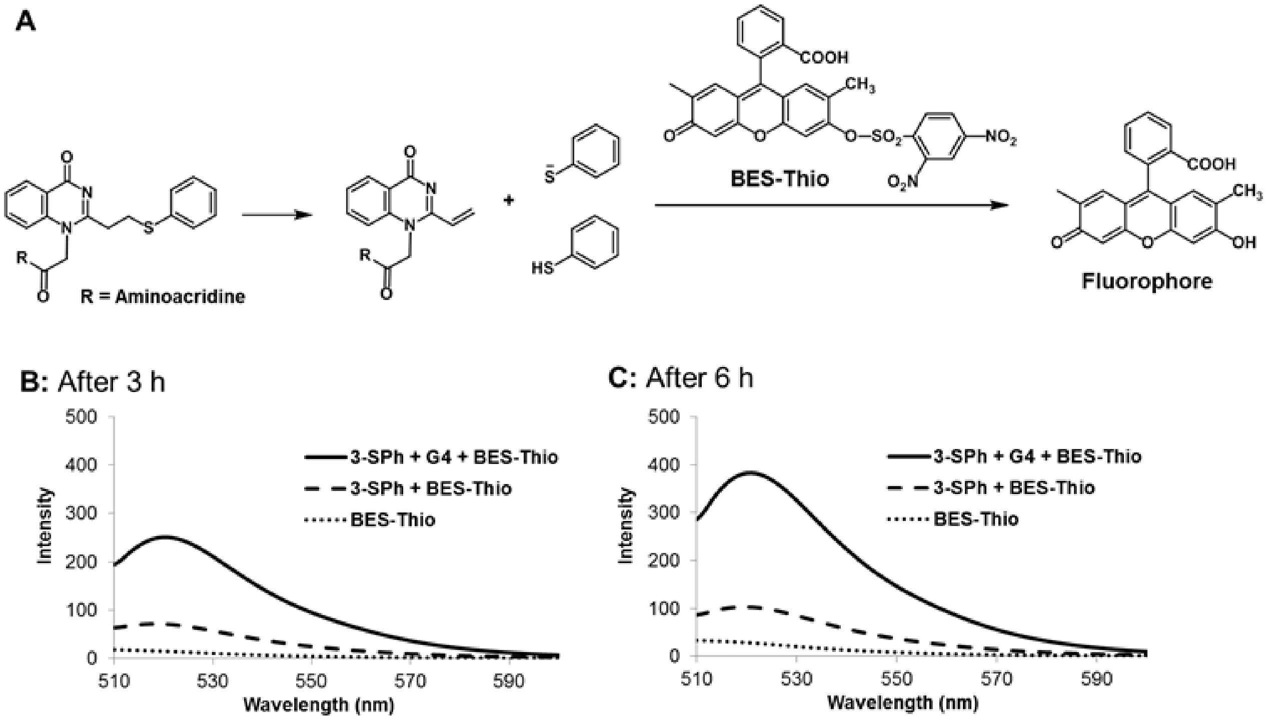

Figure S12. Detection of thiophenol by BES-Thio assay. (A) Reaction scheme of VQ-thiophenol precursor (**3-SPh**) with BES-Thio. The precursor ligand (50 μM) was incubated with BES-Thio (25 μM) in either no presence or in the presence of G-4 folded in K^+ buffer at 37°C. (B-C) Fluorescence analysis of BES-Thio reaction with **3-SPh** after 3 h (B) and 6 h (C) of incubation time.

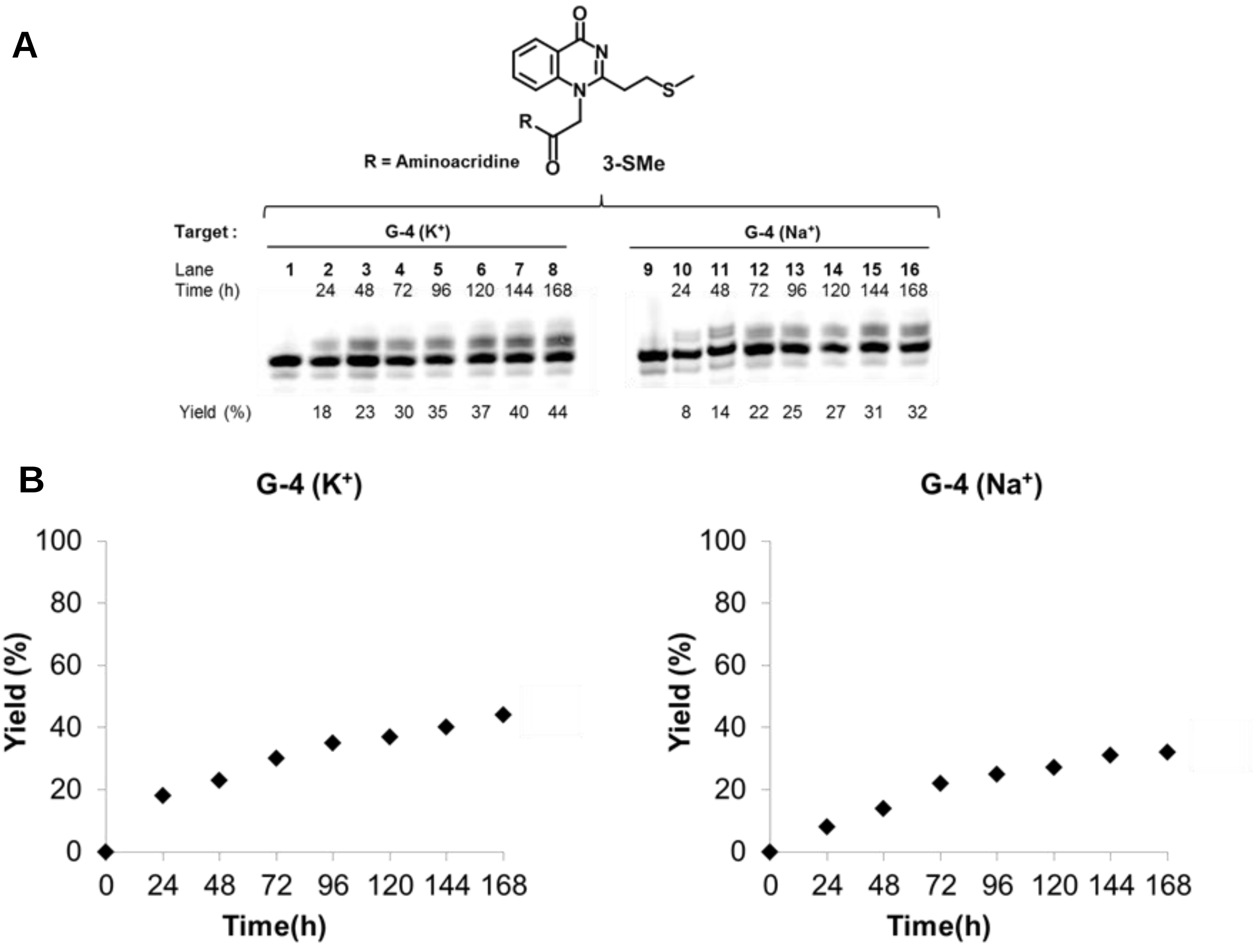

Figure S13. Alkylation by aminoacridine-VQ thiomethyl precursor (**3-SMe**). The precursor (50 μM) was incubated with G-quadruplex (G-4) DNA (2.5 μM) at 37 ^{o}C in buffer solution. G-4 potassium buffer (K^+): 100 mM KCl, 10 mM K_2HPO_4/KH_2PO_4 (pH 7.0), 1 mM K_2EDTA; G-4 sodium buffer (Na^+): 100 mM NaCl, 10 mM Na_2HPO_4/NaH_2PO_4 (pH 7.0), 1 mM Na_2EDTA. (**A**) Denaturing gel electrophoresis of the alkylation products for G-4. The electrophoresis was performed on a 16% denaturing polyacrylamide gel containing 20% formamide. (**B**) Time course of the reaction yields for **3-SMe** with G-4 folded in K^+ buffer (left) and Na^+ buffer (right).

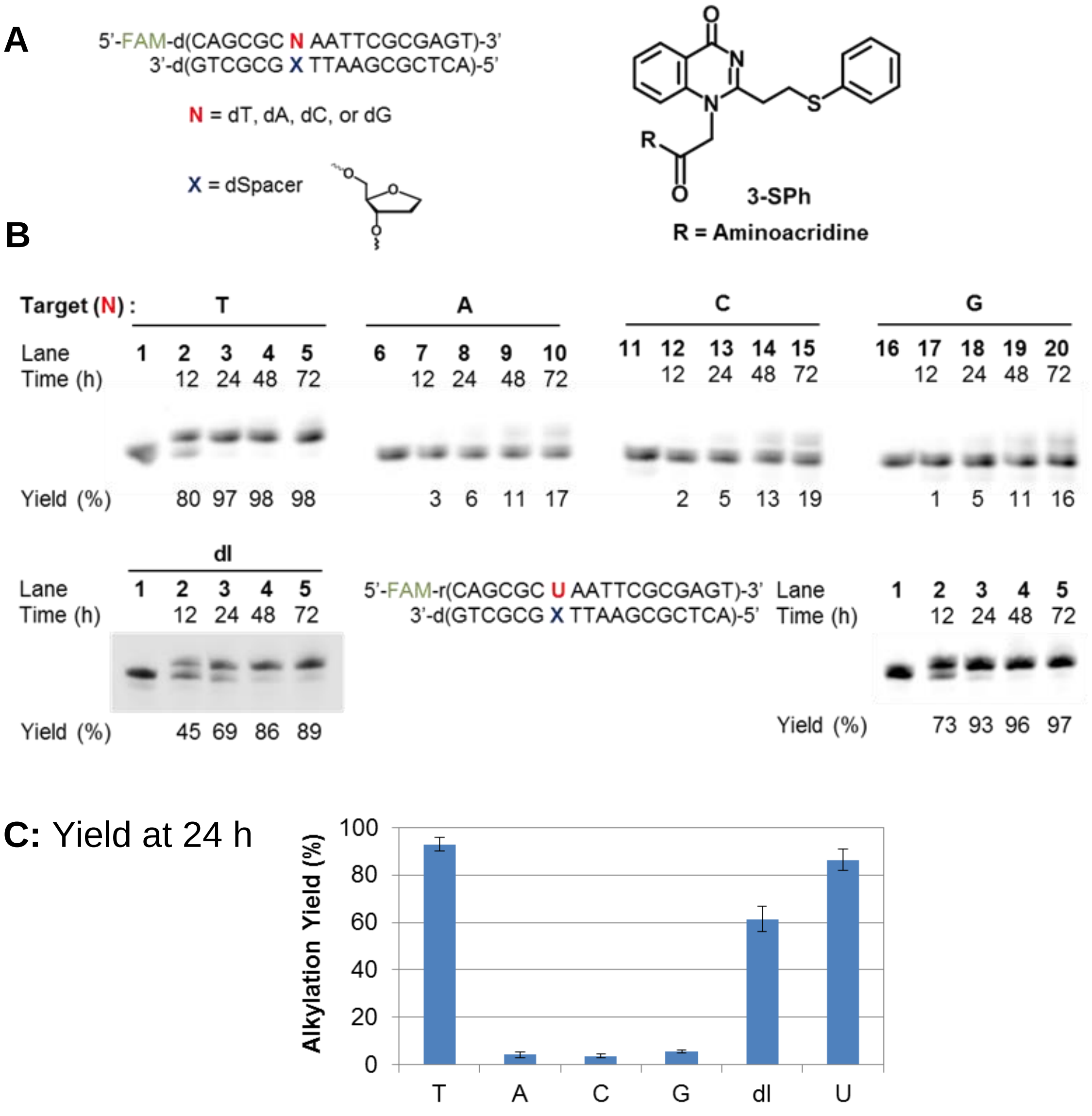

Figure S14. Alkylation of AP-site (abasic site) DNA by aminoacridine-VQ thiophenol precursor (**3-SPh**). The precursor (50 μM) was incubated with AP-site-containing DNA (2.5 μM) at 37 °C in 50 mM MES buffer (pH 7.0) containing 100 mM NaCl and 2% DMSO. (A) The The target sequences and ligand structure. (B) Denaturing gel electrophoresis of the alkylation products. The electrophoresis was performed on a 16% denaturing polyacrylamide gel containing 20% formamide. (C) Bar graph of yield (%) at 24 h (n = 3, error bars indicate standard deviation).

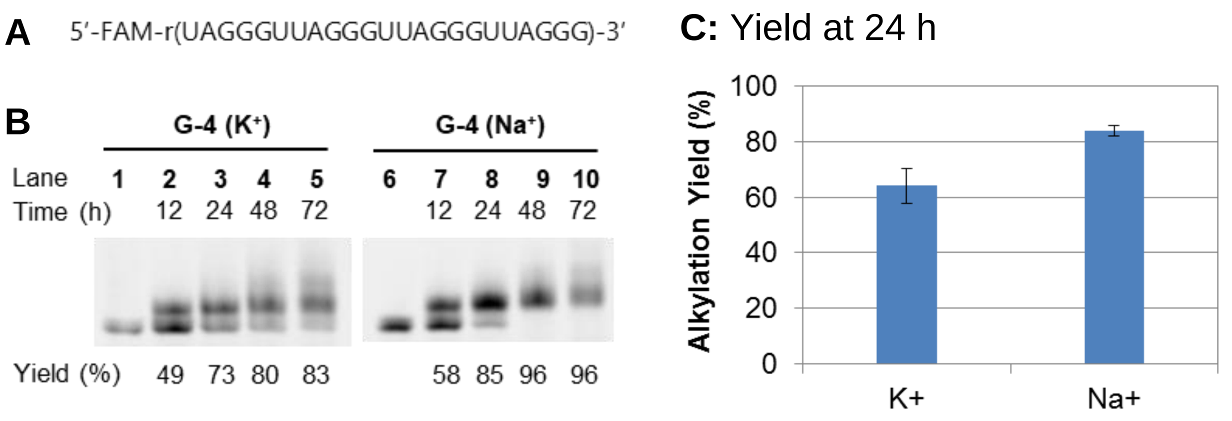

Figure S15. G-4 RNA alkylation. The precursor ligand (50 μM) was incubated with G-4 RNA (2.5 μM) at 37°C in buffer solution. G-4 potassium buffer (K^+): 100 mM KCl, 10 mM K_2HPO_4/KH_2PO_4 (pH 7.0), 1 mM K_2EDTA, G-4 sodium buffer (Na^+): 100 mM NaCl, 10 mM Na_2HPO_4/NaH_2PO_4 (pH 7.0), 1 mM Na_2EDTA. (A) The target sequence of G-4 RNA. (B) Denaturing gel electrophoresis of the alkylation products for G-4 RNA. The electrophoresis was performed on a 16% denaturing polyacrylamide gel containing 20% formamide. (C) Bar graph of yield (%) at 24 h (n = 3, error bars indicate standard deviation).

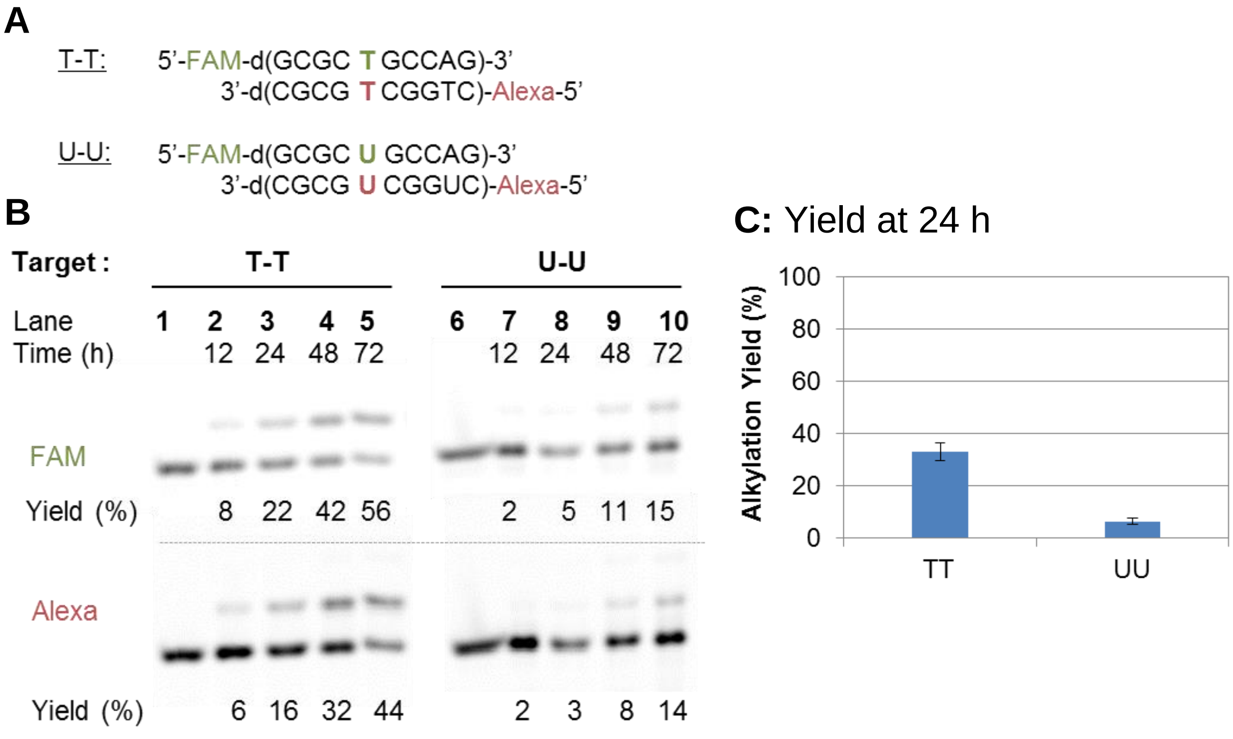

Figure S16. Alkylation of T-T and U-U mismatch by aminoacridine-VQ thiophenol precursor (**3-SPh**). The precursor (50 μM) was incubated with T-T or U-U mismatch DNA or RNA (2.5 μM) at 37 °C in 50 mM MES buffer (pH 7.0) containing 100 mM NaCl. (A) The target sequences. (B) Denaturing gel electrophoresis of the alkylation products. The electrophoresis was performed on a 16% denaturing polyacrylamide gel containing 20% formamide. (C) Bar graph of yield (%) at 24 h (n = 3, error bars indicate standard deviation).

NMR Data

Compound **6**

^{1}H NMR ($CDCl_3$, 400 MHz)

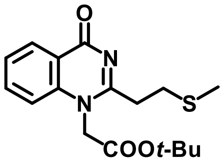

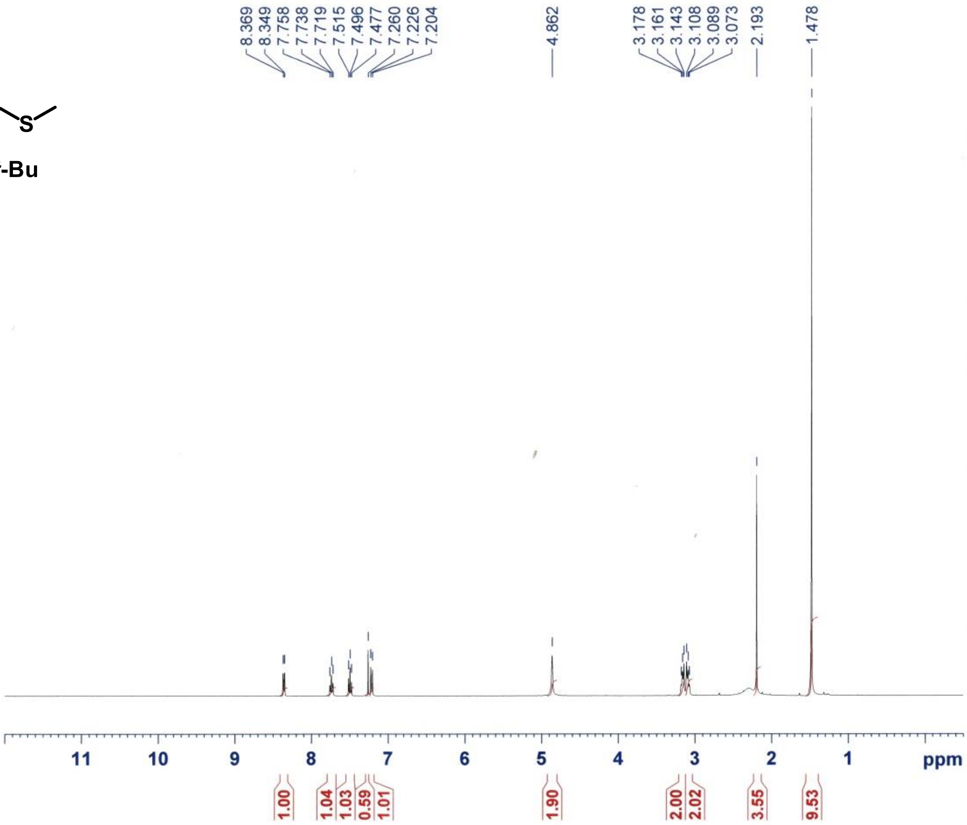

^{13}C NMR ($CDCl_3$, 125 MHz)

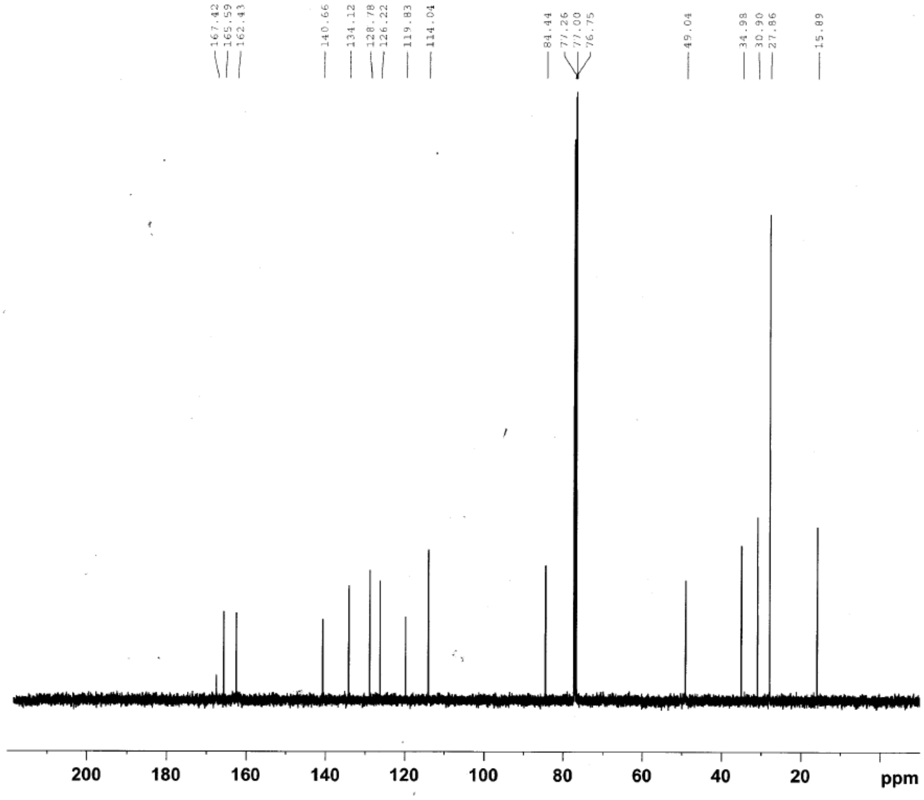

Compound **7**

^{1}H NMR (DMSO-d_6, 400 MHz)

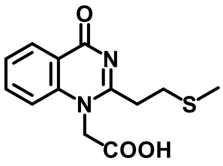

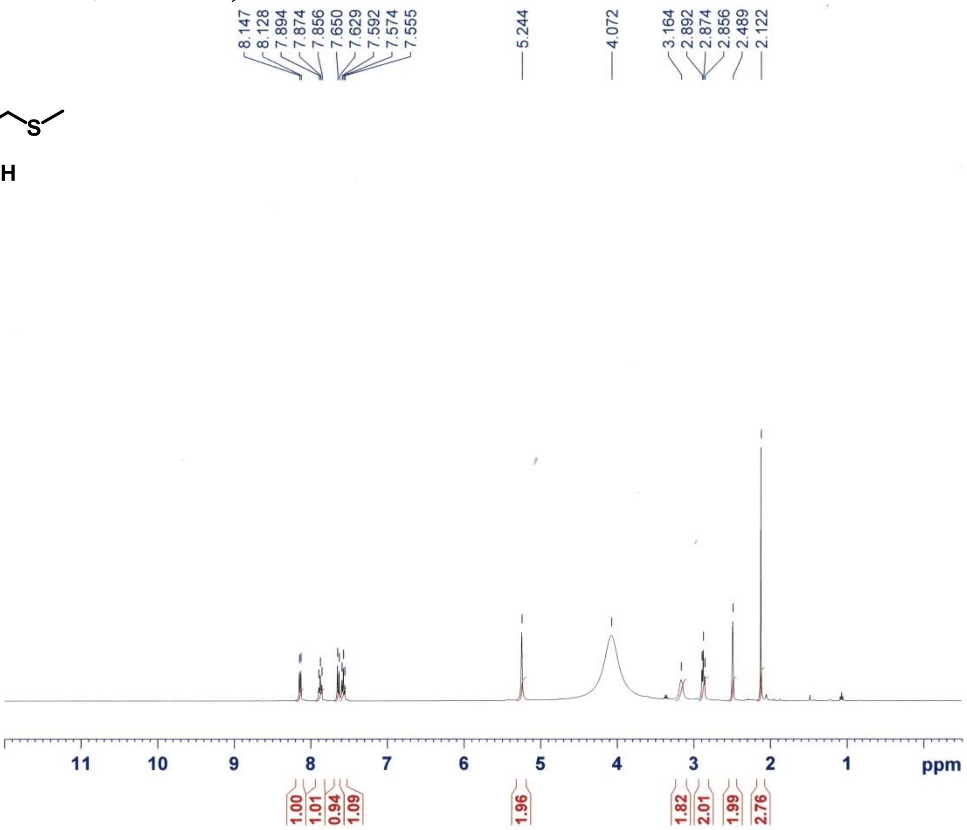

^{13}C NMR (DMSO-d_6, 125 MHz)

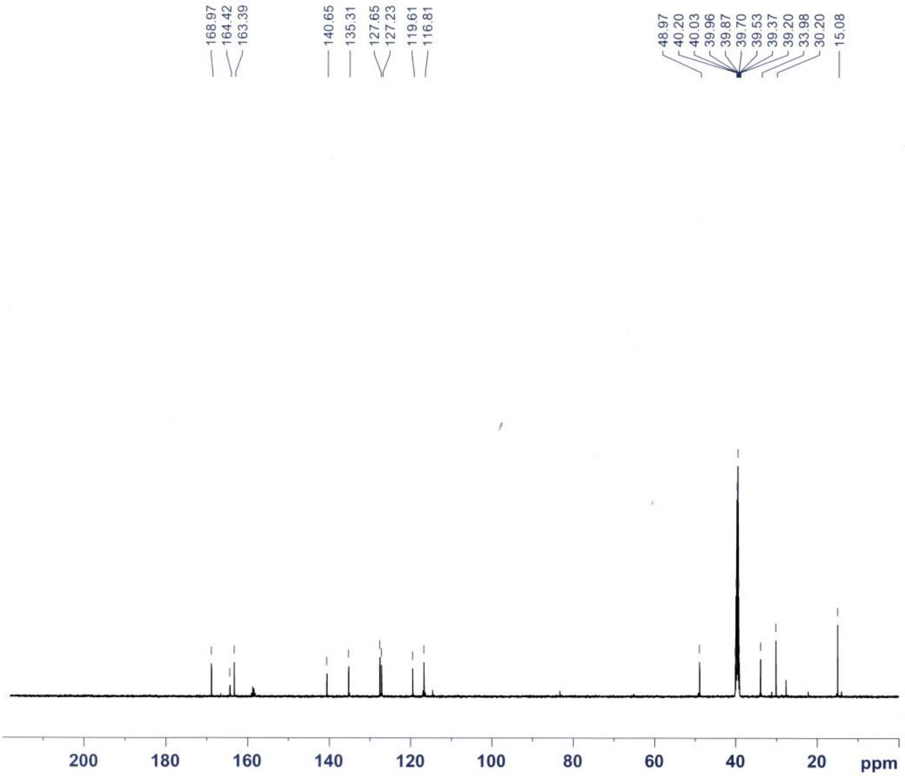

Compound **10**

^{1}H NMR ($CDCl_3$, 400 MHz)

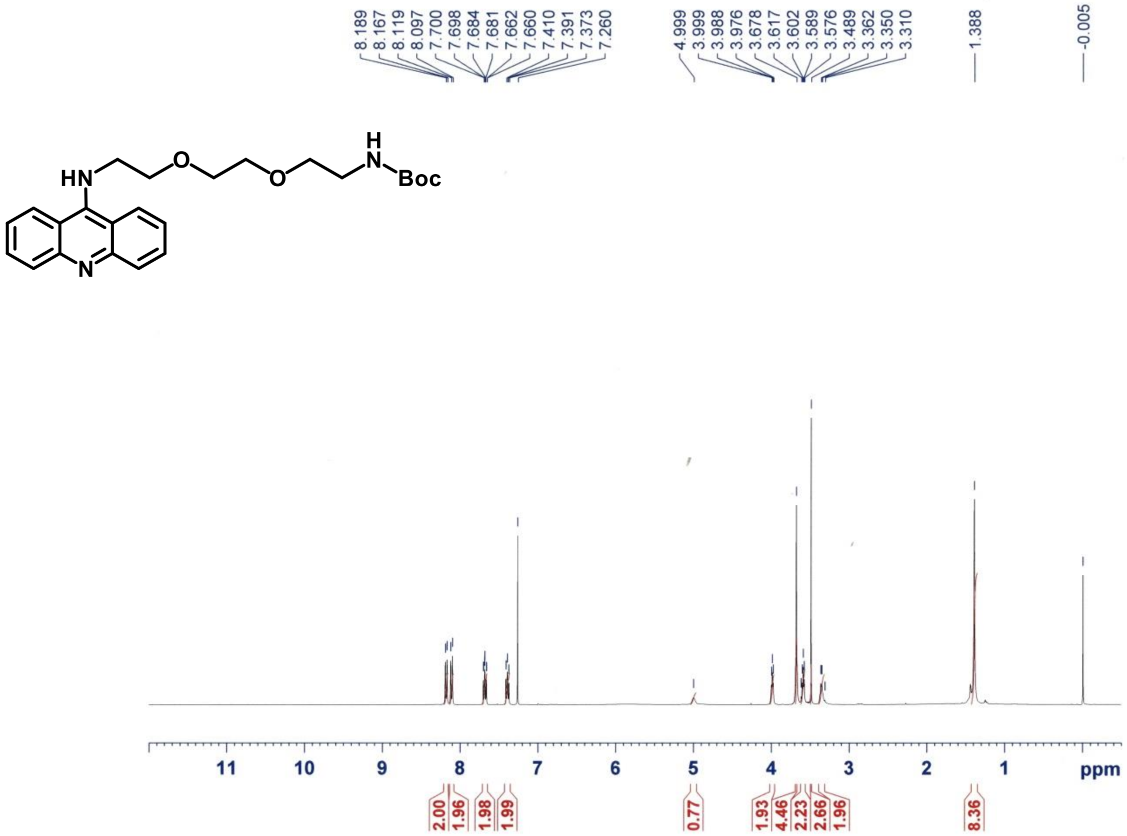

^{13}C NMR ($CDCl_3$, 125 MHz)

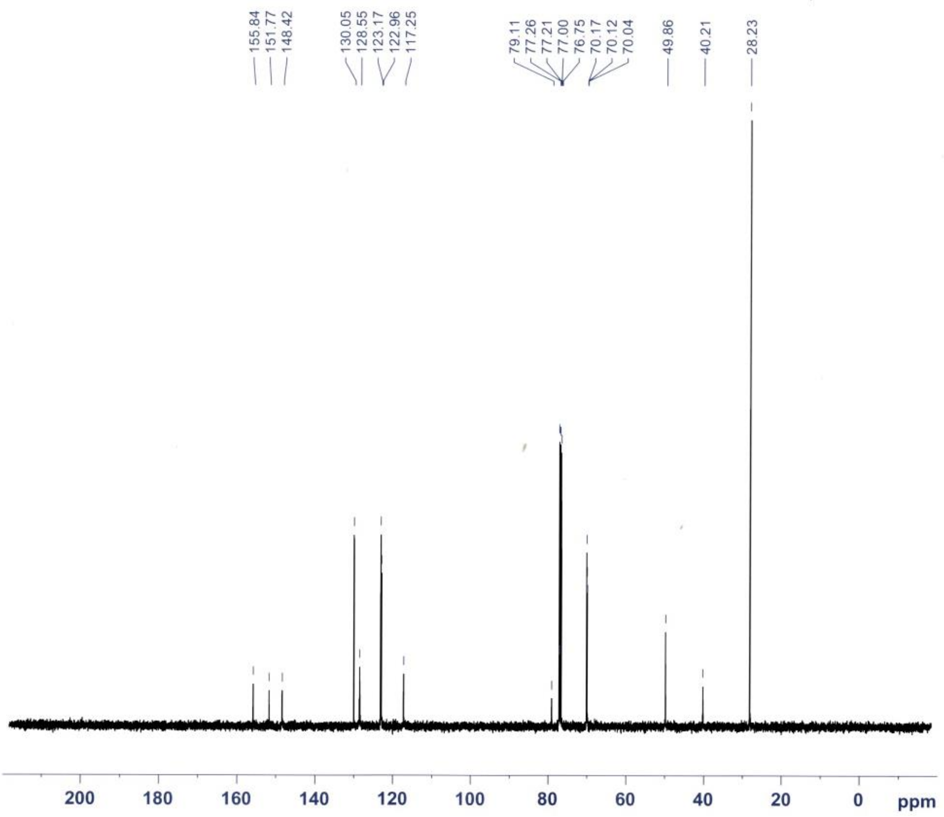

Compound **3-SMe**

^{1}H NMR (DMSO-d_6, 600MHz)

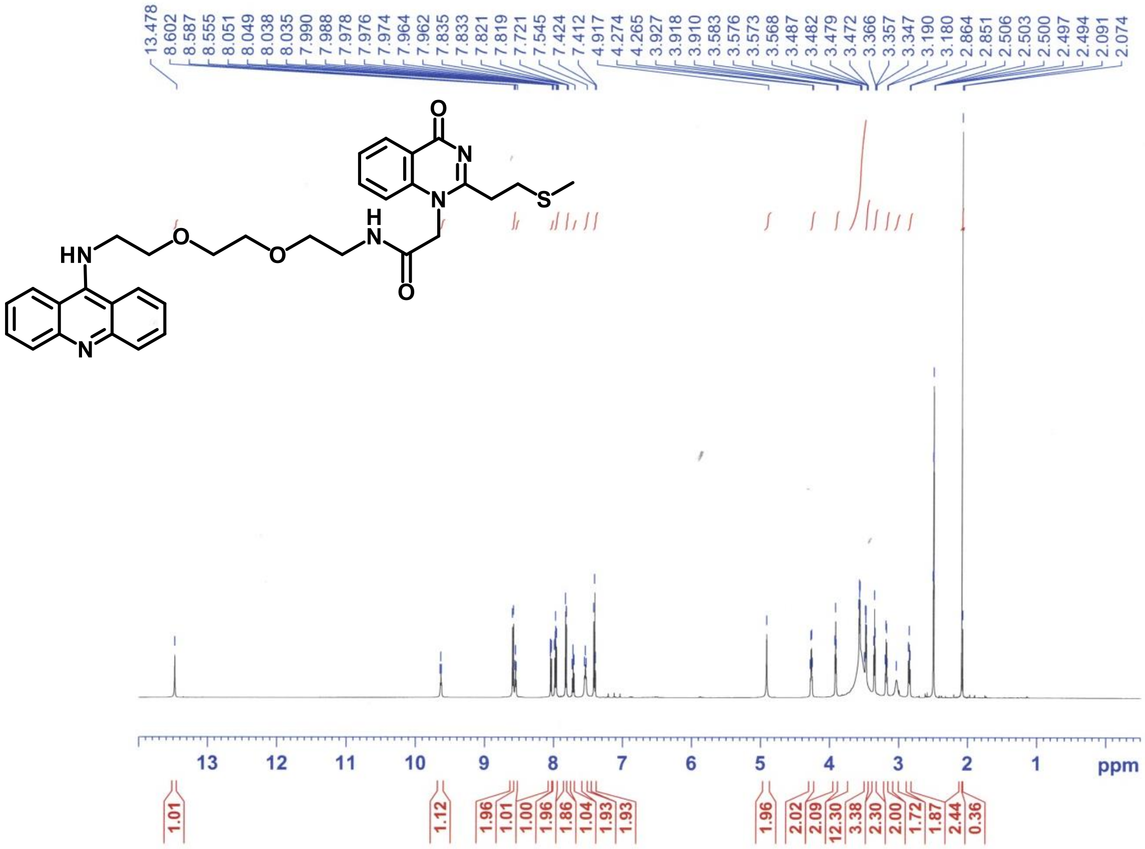

^{13}C NMR (DMSO-d_6, 150 MHz)

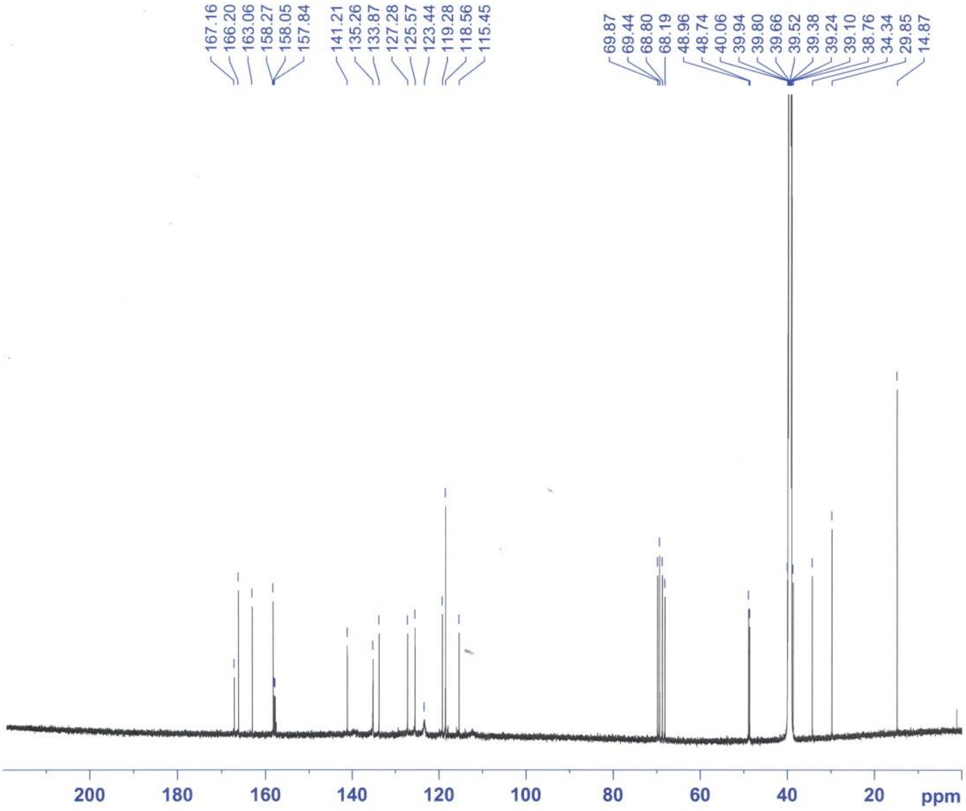

Compound **2 (3-S(O)Me)**

^{1}H NMR (DMSO-d_6, 600 MHz):

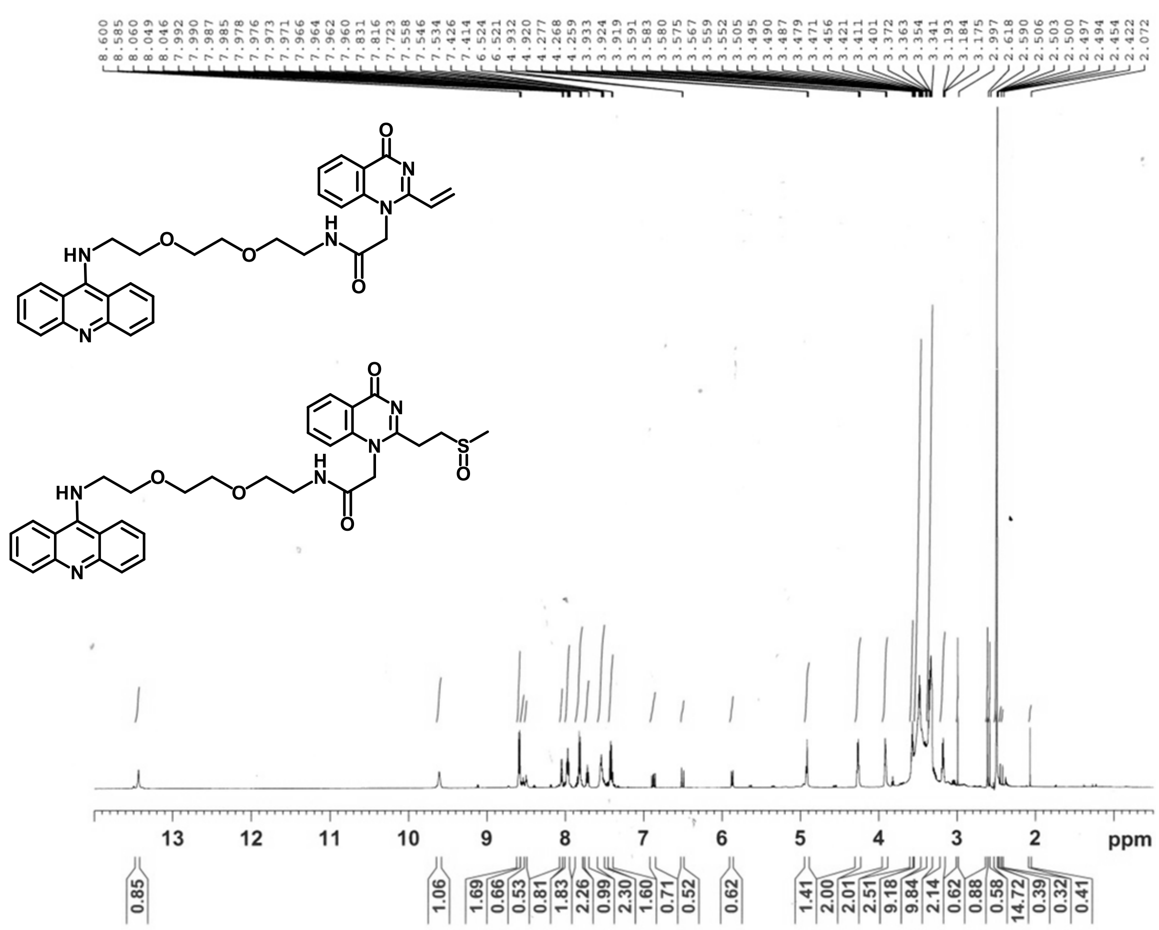

Compound **3-SPh**

^{1}H NMR (DMSO-d_6, 600 MHz):

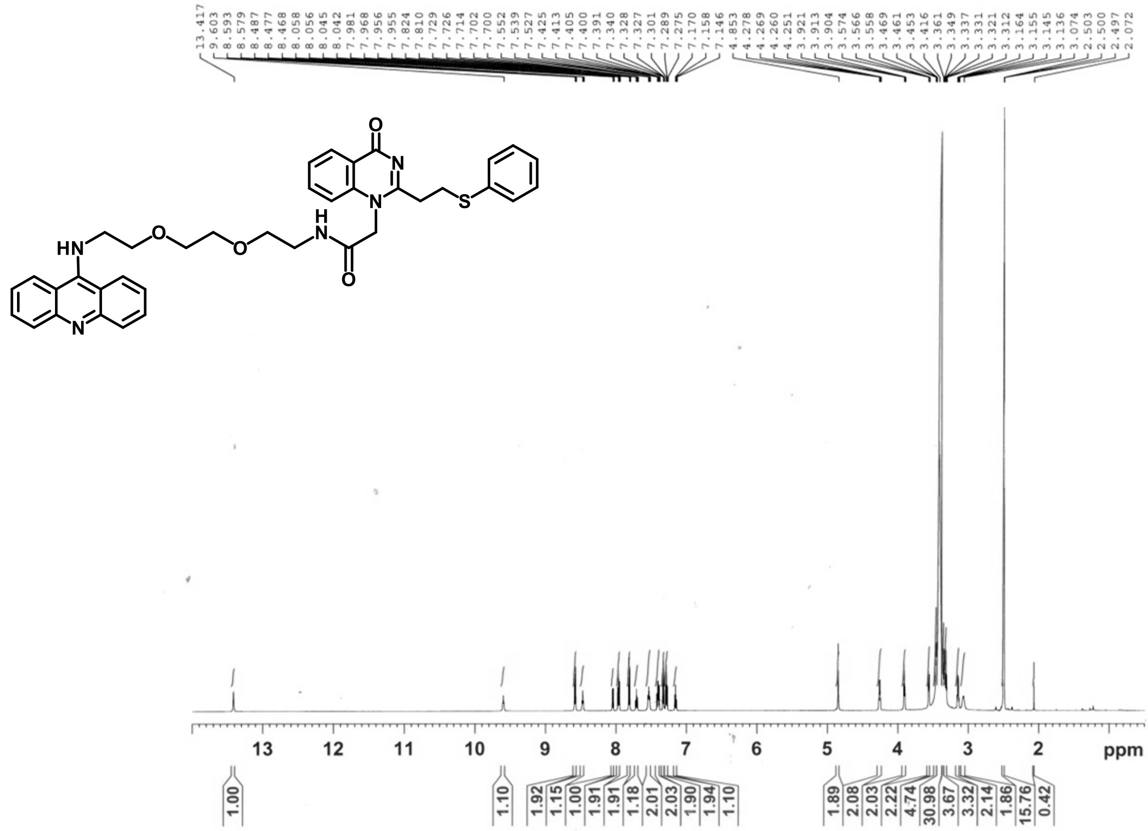

^{13}C NMR (DMSO-d_6, 150 MHz):

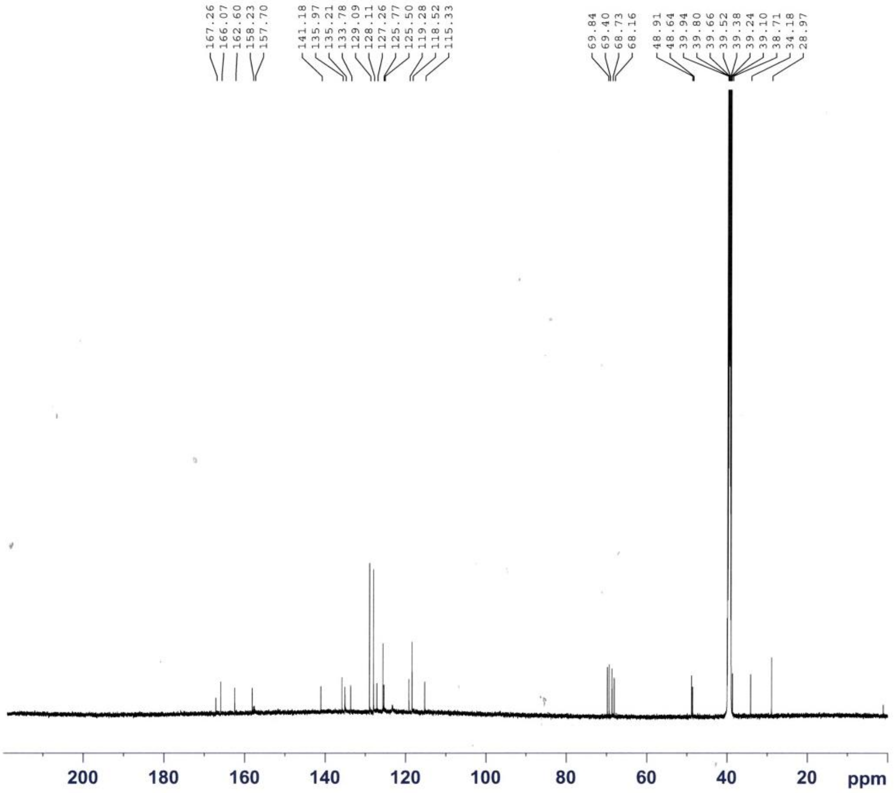

Compound VQ-dT**

^{1}H-NMR (DMSO-d_6, 600 MHz)

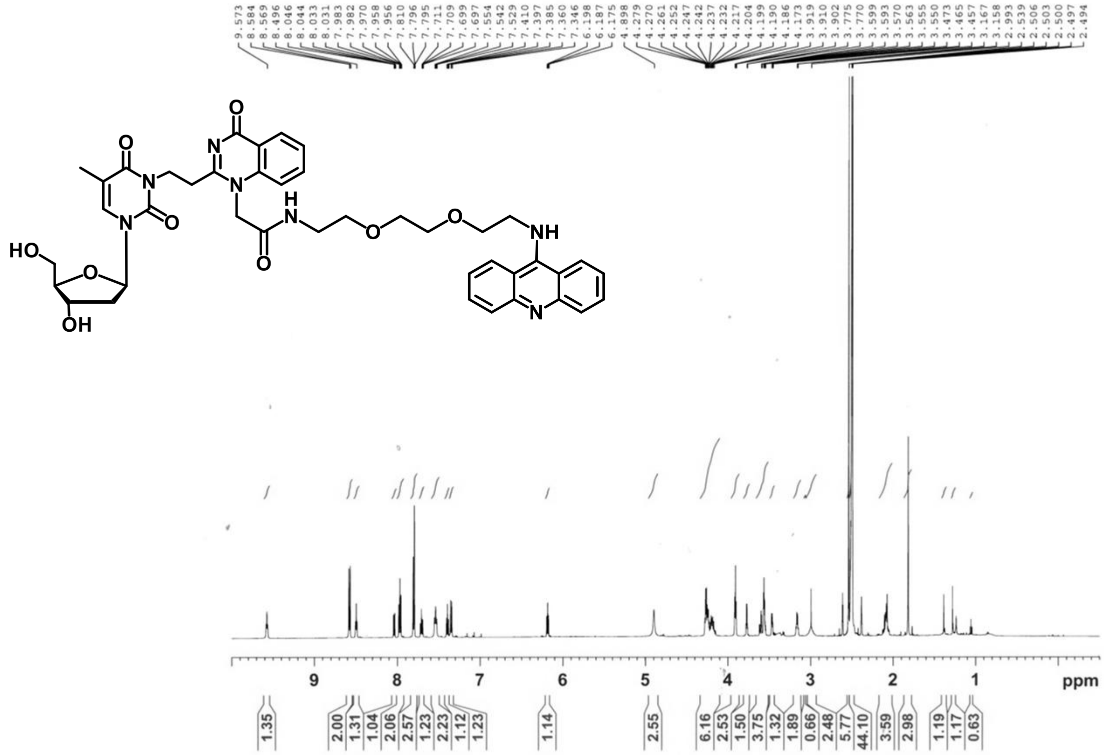

HMBC-NMR (DMSO-d_6, 600 MHz)

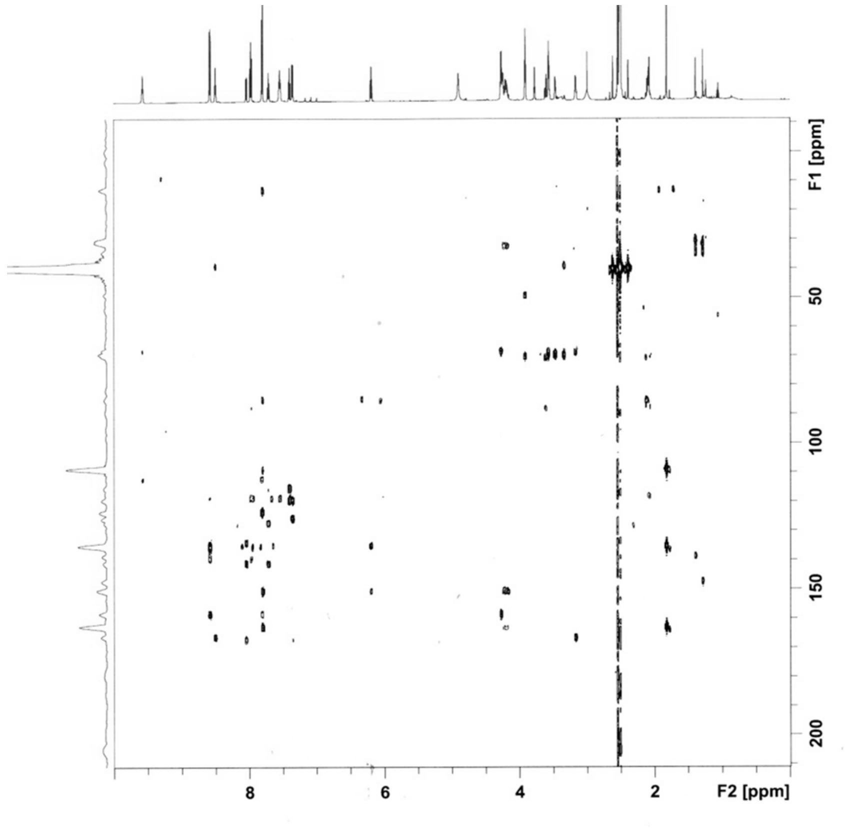

Supplement: gkz512_Supplemental_File [file gkz512_supplemental_file.pdf]
